# A genetic switch controls the production of flagella and toxins in *Clostridium difficile*

**DOI:** 10.1371/journal.pgen.1006701

**Published:** 2017-03-27

**Authors:** Brandon R. Anjuwon-Foster, Rita Tamayo

**Affiliations:** Department of Microbiology and Immunology, University of North Carolina at Chapel Hill School of Medicine, Chapel Hill, North Carolina, United States of America; Indiana University, UNITED STATES

## Abstract

In the human intestinal pathogen *Clostridium difficile*, flagella promote adherence to intestinal epithelial cells. Flagellar gene expression also indirectly impacts production of the glucosylating toxins, which are essential to diarrheal disease development. Thus, factors that regulate the expression of the *flgB* operon will likely impact toxin production in addition to flagellar motility. Here, we report the identification a “flagellar switch” that controls the phase variable production of flagella and glucosylating toxins. The flagellar switch, located upstream of the *flgB* operon containing the early stage flagellar genes, is a 154 bp invertible sequence flanked by 21 bp inverted repeats. Bacteria with the sequence in one orientation expressed flagellum and toxin genes, produced flagella, and secreted the toxins (“*flg* phase ON”). Bacteria with the sequence in the inverse orientation were attenuated for flagellar and toxin gene expression, were aflagellate, and showed decreased toxin secretion (“*flg* phase OFF”). The orientation of the flagellar switch is reversible during growth *in vitro*. We provide evidence that gene regulation via the flagellar switch occurs post-transcription initiation and requires a *C*. *difficile*-specific regulatory factor to destabilize or degrade the early flagellar gene mRNA when the flagellar switch is in the OFF orientation. Lastly, through mutagenesis and characterization of flagellar phase locked isolates, we determined that the tyrosine recombinase RecV, which catalyzes inversion at the *cwpV* switch, is also responsible for inversion at the flagellar switch in both directions. Phase variable flagellar motility and toxin production suggests that these important virulence factors have both advantageous and detrimental effects during the course of infection.

## Author summary

*Clostridium difficile* is a bacterial pathogen that causes antibiotic associated diarrheal disease. Colonization of host tissues is a prerequisite step to disease development during bacterial infection. *C*. *difficile* produces flagella, which are proteinaceous structures on the bacterial surface that confer motility and participate in adherence to the host intestine. SigD, a regulator that coordinates flagellar gene expression, also activates expression of the toxin genes in *C*. *difficile*. Therefore, mechanisms controlling expression of flagellar genes, including *sigD*, will likely impact the severity of *C*. *difficile* infection given the impact on toxin production. In this body of work, we identified a genetic ON/OFF switch, which we term the “flagellar switch”, encoded upstream of the flagellar genes. The orientation of the flagellar switch determines whether or not *C*. *difficile* produce flagella, engage in swimming motility, and secrete toxins. We identified the enzyme that catalyzes inversion of the flagellar switch. Inactivation of the corresponding gene resulted in bacteria with the flagellar switch locked in the either the ON or OFF orientation, with concomitant effects on flagellum and toxin biosynthesis. The flagellar switch may represent a new regulatory strategy to coordinately control virulence determinants independent of previously described regulators.

## Introduction

*Clostridium difficile*, a Gram-positive, spore-forming obligate anaerobe, is the leading cause of nosocomial disease in the North America, Europe and Australia [1,2]. The Centers for Disease Control and Prevention list *C*. *difficile* infections (CDI) as an urgent threat related to the use of antibiotics [3]. Antibiotic use perturbs the gastrointestinal microbiota that normally protects against CDI [1,4]. The rates of recurrence and mortality associated with CDI have increased in part due to the emergence of epidemic-associated strains with enhanced sporulation rates and toxin production [1,5,6]. The *C*. *difficile* PCR ribotype 027 group is associated with greater odds of diarrheal disease severity, outcome, and death compared to many other PCR ribotypes [7]. Therefore, an understanding of bacterial physiology and genetics in *C*. *difficile* 027 ribotypes could reveal unique therapeutic or diagnostic targets to ameliorate severe CDI.

*C*. *difficile* is primarily transmitted as metabolically dormant spores, which germinate into actively growing vegetative cells in response to bile salts such as glycine-conjugated taurocholate [8–10]. CDI ranges in severity from mild self-limiting diarrhea to fulminant colitis characterized by neutrophil infiltration into the lamina propria, erosion of crypts and goblet cells, and extensive epithelial tissue damage. Diarrheal disease is associated with strains that produce the glucosylating toxins TcdA and/or TcdB [11,12]. Both TcdA and TcdB glucosylate Rho and Rac GTPases in host cells to promote actin depolymerization, destruction of tight junctions at the mucosal barrier, and inflammation [13–16]. The glucosylating toxins are necessary for diarrheal disease in both the mouse and hamster models of infection, with TcdB playing a more prominent role [11,12,17].

Colonization is a requisite step to diarrheal disease, and the bacterial surface structures, such as flagella, that participate in colonization are an active area of study. The flagellar apparatus confers motility and contributes to adherence, colonization, and disease in *C*. *difficile* [18–20]. Flagellum biosynthesis genes are conserved in most sequenced *C*. *difficile* strains [19,21]. As in other bacterial species, flagellar gene transcription occurs in a hierarchical order to ensure proper protein assembly and to conserve energy. The early stage flagellar operon contains genes for assembly of the basal body. SigD (σ^D^), the flagellar alternative sigma factor, is encoded in the early stage flagellar operon and activates the transcription of the late stage operons [22,23]. At least four operons contain late stage flagellar genes involved in assembly of the flagellar hook, filament, and cap, and for post-translational modification of the flagellar filament [18,22,24–26]. Flagellar arrangement is peritrichous in most strains [18,27–31].

The flagellar apparatus contributes to *C*. *difficile* fitness during gastrointestinal infection in a ribotype-dependent manner [20]. In *C*. *difficile* 630Δ*erm* (ribotype 012), flagella are dispensable for adherence to cultured epithelial cells and colonization in the murine model [31,32]. In contrast, in the epidemic-associated *C*. *difficile* strain R20291 (ribotype 027), flagellar filaments promote adherence and colonization *in vitro* and during infection of mice [31]. Flagellar motility is not required for adherence in this strain, as a MotB mutant that produces flagellar filaments with a nonfunctional motor displays wild type adherence [31]. Interestingly, mice infected with the R20291 *fliC* mutant succumb to infection, whereas mice infected with the parental strain do not [31]. The R20291 *fliC* mutant has altered expression of genes involved in motility, membrane transport, sporulation, and metabolism *in vitro* [33], which may explain the enhanced virulence in mice. Post-translational modification of flagella also contributes to colonization kinetics in a mouse relapse model of infection in M68, an 017 ribotype strain [24]. In the hamster model, *C*. *difficile* 630Δ*erm* strains with a mutation in several early stage flagellar genes showed reduced virulence, whereas *C*. *difficile* with mutations in the late stage flagellar genes *fliC* and *fliD* generally showed increased virulence [22,32]. Several groups have observed that a 630Δ*erm fliC* mutant produces more toxin, which may contribute to the increased virulence of the mutant [22,31,32]. The animal model-dependent differences in virulence phenotypes of the flagellar gene mutants may be attributed to the greater sensitivity of hamsters to the glucosylating toxins [34], and to higher spore germination rates of *C*. *difficile* spores in hamsters than in mice [35].

Notably, the expression of the glucosylating toxin genes is linked to flagellar gene expression in *C*. *difficile* [20,36]. TcdA and TcdB are encoded on a Pathogenicity Locus (PaLoc) along with the TcdR sigma factor that positively regulates *tcdA* and *tcdB* [37], the anti-sigma factor TcdC suggested to inhibit TcdR function [38–41], and the TcdE holin-like protein suggested to be involved in toxin export [42,43]. Preliminary studies suggested that σ^D^ affects the expression of the glucosylating toxin genes [22]. Subsequent work identified a σ^D^ consensus sequence in the *tcdR* promoter [23,44]. Overexpression of *sigD* in *C*. *difficile* increases toxin production in a TcdR-dependent manner [44], and recombinant σ^D^ with RNA polymerase directly binds the *tcdR* promoter [23]. Taken together, these studies highlight a regulatory link between virulence factors critical to host colonization and to disease symptom development in *C*. *difficile*, similar to other diarrheal bacterial pathogens, such as *Vibrio cholerae* and *Campylobacter jejuni* [45,46].

*C*. *difficile* flagellin and the glucosylating toxins stimulate pathogen recognition receptors [47–49], which promote pathogen clearance mechanisms, and therefore their production must be subject to precise regulation to avoid host recognition. In addition to σ^D^, several transcriptional regulators modulate toxin production in *C*. *difficile*, potentially via regulation of flagellar gene expression. Spo0A, SigH, and RstA inhibit expression of the flagellar and toxin genes [50–53], while Agr quorum sensing and Hfq are positive regulators [29,54]. The exact mechanisms by which these regulators control flagellar and toxin gene expression are largely unknown.

Cyclic diguanylate (c-di-GMP) is a nucleotide second messenger that controls a multitude of bacterial processes, such as flagellar motility, biofilm formation, and virulence factor expression [55–58]. In *C*. *difficile*, c-di-GMP regulates swimming motility, cytopathicity, Type IV-pilus dependent surface motility and biofilm formation [30,44,59,60]. Specifically, elevated c-di-GMP inhibits flagellar gene expression and swimming motility [59], and also negatively regulates toxin gene expression as a result of reduced *sigD* transcription [44]. Flagellar gene regulation occurs via a c-di-GMP sensing riboswitch, Cd1, located in the 5’ untranslated region of the early stage flagellar operon [61]. c-di-GMP binding to Cd1 causes premature transcription termination, resulting in a truncated transcript of 160 nt of the 5’ untranslated region [62].

Here, we describe the identification and characterization of an additional *cis*-acting regulatory element that mediates phase variable expression of flagellar genes and, consequently, the toxin genes. We identified a flagellar switch consisting of 154 bp flanked by 21 bp imperfect inverted repeats, between the Cd1 riboswitch and the first open reading frame in the early stage flagellar operon. The orientation of the flagellar switch controls downstream flagellar gene expression, including *sigD*, and therefore production of flagella and swimming motility. Furthermore, the flagellar switch affects transcription of the toxin genes by way of σ^D^, impacting the production of the glucosylating toxins and the cytotoxicity of *C*. *difficile*. We provide evidence that regulation through the flagellar switch occurs post-transcription initiation. Lastly, we identified RecV, which also controls phase variation of the cell wall protein CwpV, as the recombinase responsible for inversion at the flagellar switch in both directions. Together these findings indicate that flagellar motility and the production of toxins are subject to phase variation in *C*. *difficile*, thus identifying an additional level of regulation of these linked processes. Flagellar and toxin phase variation in *C*. *difficile* may confer an advantage to the bacterium during infection of the gastrointestinal tract as a bet hedging strategy to either promote colonization and inflammation as certain tissue sites or persist and evade host immune stimulation.

## Results

### *In silico* identification of a flagellar switch upstream of the early flagellar biosynthesis operon

**Fig 1:**
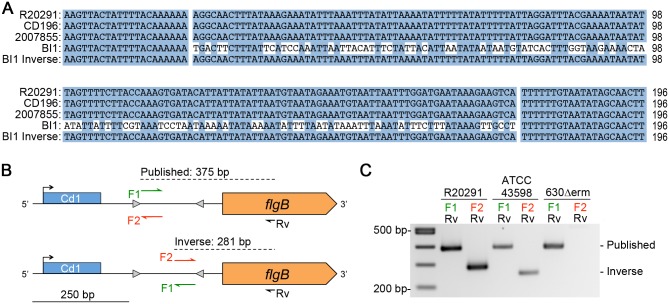
Evidence for DNA inversion at the flagellar switch. (A) Nucleotide sequences corresponding to the 5’ UTR of the *flgB* operon from genome sequences available for PCR ribotype 027 strains were aligned using Clustal Omega. Shown are the regions corresponding to the putative flagellar switch and flanking imperfect inverted repeats. For strain BI1 “inverse”, the alignment was repeated after replacing the putative switch with its reverse complement. Identical nucleotides are indicated with blue shading. (B) Diagram of the PCR strategy used to detect the putative flagellar switch orientation. The primer names and sequences used for each strain are listed in the S2 Table. The predicted product sizes are based on R20291 sequence. (C) Orientation-specific PCR products for the flagellar switch from three *C*. *difficile* strains representing three ribotypes (R20291, 027, NCBI Accession No FN545816.1; ATCC43598, 017, NCBI sequence read archive SRX656590 [115]; 630Δ*erm*, 012, NCBI Accession No. EMBL:LN614756 [116]).

Soutourina, *et*. *al*., identified a single transcriptional start site (TSS) for the early flagellar operon in *C*. *difficile* strain 630Δ*erm*, located nearly 500 nucleotides (nt) upstream of the start codon for *flgB*, the first gene in this operon [62]. The *flgB* operon is also termed the “F3 locus” elsewhere [21]. The promoter for the *flgB* operon contains a σ^A^ consensus sequence [62]. The first 160 nt of the 5’ untranslated region (UTR) of the *flgB* operon contains a class I (GEMM) c-di-GMP riboswitch, previously named Cd1 [61]. Binding of c-di-GMP to Cd1 causes premature transcription termination, preventing flagellar gene transcription and inhibiting swimming motility [59,61,62]. Notably, an additional 338 nt lie between the Cd1 termination site and the start codon of *flgB*. We speculated that the remaining 338 nt of the 5’ UTR contains an additional regulatory element that controls expression of the *flgB* operon. To explore this possibility, we used Clustal Omega [63] to generate multiple sequence alignments and examine the 5’ UTR of the *flgB* operon for regions of reduced sequence identity. Assuming the TSS is conserved in all strains, we found that all *C*. *difficile* genome sequences currently available through NCBI contain a 498 nt 5’ UTR, except 630 which has a 496 nt 5’ UTR. Strains of the 027 ribotype, which is associated with epidemics of CDI, were used for further analysis: CD196 (NCBI Accession No. FN538970.1), R20291 (FN545816.1), 2007855 (FN665654.1), and BI1 (FN668941.1) [19,64,65]. The 5’ UTRs of three strains exhibited 100% sequence identity, but BI1 had only 66.2% identity in a 154 bp region (Fig 1A). Upon further scrutiny of the region, we identified near perfect 21 bp inverted repeats flanking the 154 bp region: 5’- AAGTT(A/G)CTAT(A/T)TTACAAAAAA-3’ (Left inverted repeat). The presence of a sequence flanked by inverted repeats located upstream of genes encoding an immunostimulatory surface structure suggested a regulatory mechanism involving phase variation by site specific DNA recombination. These features reflect a regulatory mechanism employed by numerous mucosal bacterial pathogens to stochastically control the expression of cell surface structures that may be immunostimulatory, therefore promoting immune evasion and persistence in a host [66]. We reasoned that the reduced sequence identity for BI1 at the 154 bp region could be due to DNA inversion between the inverted repeats. To test this, we repeated the multiple sequence alignment using the antiparallel, “inverse” sequence for the 154 bp sequence for BI1 and observed restoration of 100% identity to the other ribotype 027 sequences (Fig 1A). Based on these *in silico* findings, we hypothesized that the 154 bp sequence undergoes inversion via site-specific recombination, and we term this the “flagellar switch” herein.

### The flagellar switch undergoes DNA inversion

If the 154 bp sequence is capable of undergoing DNA inversion, we expect to detect the flagellar switch in both of the orientations in *C*. *difficile*, at least under some growth conditions. To test this, we used an orientation-specific PCR assay to detect and differentiate between the two orientations of the putative flagellar switch in multiple *C*. *difficile* strains grown in liquid medium (Fig 1B) [67,68]. We use “published orientation” to refer to the sequence present in the indicated published genome for the given strain, and “inverse orientation” to refer to a sequence with an inversion between the inverted repeats. A common reverse primer, Rv, complementary to the *flgB* coding sequence was used in all PCRs (Fig 1B, black). To detect the flagellar switch in the published orientation, we used a ribotype-specific forward primer F1 that anneals immediately 3’ of the left inverted repeat (LIR) (Fig 1B, green). To detect the flagellar switch in the inverse orientation, we used a forward primer F2 that anneals immediately 5’ of the right inverted repeat (RIR) (Fig 1B, red). Primers F1 and F2 are reverse-complementary. The primer pairs yield different products of different sizes depending on the orientation of the template sequence, and the sizes vary somewhat depending on the strain. We detected PCR products for both the published and inverse orientations for R20291 (FN545816.1) and ATCC 43598 (017 ribotype, sequence read archive SRX656590) (Fig 1C). DNA sequencing of the PCR products confirmed the orientation of the template sequences. Furthermore, DNA sequencing suggests that DNA strand exchange for recombination would have to occur at or after position 12 of the IRs, because the 6^th^ and 11^th^ nucleotides that are not conserved do not change. We were unable to detect a PCR product for the inverse orientation for 630*Δerm* (Fig 1C), a commonly used laboratory-adapted strain. Unlike the other published *C*. *difficile* genomes, 630Δ*erm* and its parental strain, 630, have shorter 20 bp IRs with an adenosine absent from the 3’ end of the LIR and a thymidine absent from the 5’ end of the RIR. The length of IRs can impact DNA inversion [68], which may explain the lack of an inverse product for 630Δ*erm*. Collectively, these data indicate that at least some *C*. *difficile* strains grow in broth culture as a heterogeneous population of bacteria with the flagellar switch in the published or inverse orientation, supporting our hypothesis that the flagellar switch undergoes site-specific recombination. Based on the previously identified inversion sites in the *C*. *difficile* genomes [69], we designate the flagellar switch site as Cdi4.

### Quantifying the frequency of flagellar switch orientation in enriched flagellar phase variant populations

**Fig 2:**
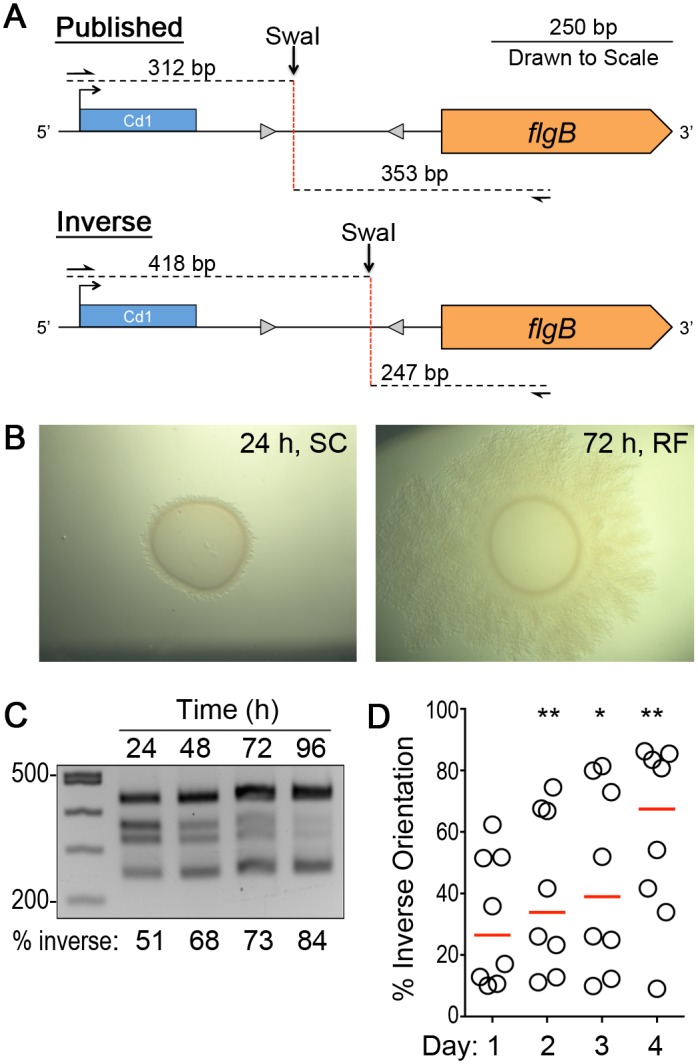
Enrichment for flagellar phase variant populations. (A) Diagram of the asymmetric PCR-digestion method used to determine the proportions of bacteria in the population with the putative flagellar switch in each orientation. (B) Representative images of *C*. *difficile* R20291 colonies after 24 hours of growth, which are circular with smooth edges (left), and after 72 hours of growth, which are rough with filamentous edges (right). (C) Asymmetric PCR-digestion assay showing the proportions of bacteria with the flagellar switch in each orientation within colonies collected every 24 hours for four days. Below each lane is the percentage of the colony population in which the flagellar switch is in the inverse orientation, calculated by comparing relevant band intensities to the total normalized to a standard curve. (D) Quantitative measurements of the percentage of bacteria in each colony with the switch in the inverse orientation over the course of four days, with relevant band intensities normalized to a standard curve (S1 Methods). Each circle represents one of eight biological replicates monitored over time. * *p* < 0.05, ** *p* < 0.01 by one-way ANOVA and Dunnett’s multiple comparisons test.

We optimized an unbiased asymmetric PCR-digestion assay to distinguish the orientation of the switch and determine the relative proportions of each in the population (Fig 2A) [67,68,70]. One pair of primers that flank the flagellar switch amplify a region encompassing the 5’ UTR of the *flgB* operon, yielding a 665 bp product that was subjected to digestion with the restriction enzyme SwaI. The presence of a single SwaI restriction site in the 154 bp flagellar switch resulted in two fragments of different sizes depending on the orientation of the sequence (Fig 2A). If the flagellar switch is in the published orientation, we expect fragments of 312 and 353 bp; if the switch is in the inverse orientation, we expect fragments of 418 and 247 bp. All four products are expected for a mixed population.

Spore preparations of *C*. *difficile* R20291, which contain a mix of bacteria with respect to flagellar switch orientation (S1 Fig), were plated on BHIS agar supplemented with the germinant taurocholate. Germinated colonies were collected and spotted onto standard BHIS agar. We observed that over 96 hours, the R20291 colony morphology changed from smooth and circular to a spreading, filamentous colony (Fig 2B). Genomic DNA was isolated from replicate colonies every 24 hours, and the asymmetric PCR-digestion assay was performed after all samples were processed. Over time, the abundance of the SwaI digested products indicating the inverse orientation increased (Fig 2C). The proportions of bacteria with the switch in the two orientations were quantified by measuring the pixel intensities of the bands, normalized to a standard curve generated with titrated amounts of SwaI-digested DNA template for the published and inverse orientations [71]. The proportion of the bacteria with the flagellar switch in the inverse orientation significantly increased after 48 hours (median of 33.8%, *P* < 0.005), 72 hours (39.0%, *P* < 0.05), and 96 hours (67.4%, *P* < 0.05) compared to 24 hours (26.4%) (Fig 2D). These data provide evidence of DNA inversion and suggest that growth on a solid agar surface favors the accumulation of *C*. *difficile* with the flagellar switch in the inverse orientation.

**Fig 3:**
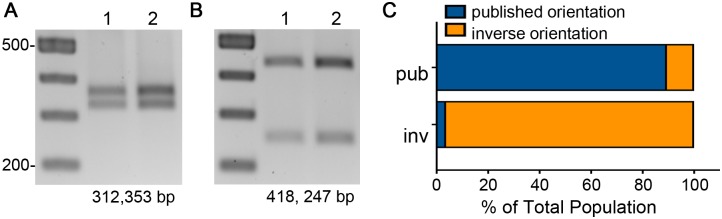
Assessing the purity of enriched populations. (A, B) Asymmetric PCR-digestion assay of genomic DNA to determine the orientation of the flagellar switch in individual colonies. Bacteria from individual colonies contain the switch predominantly in the published orientation (A) or the inverse orientation (B). Two representatives of each are shown. (C) Quantitative PCR results of the flagellar switch orientation in enriched phase variant populations (published, n = 9; inverse, n = 10). The *ΔΔCt* method was used to determine the relative DNA copies of the flagellar switch orientation in enriched populations relative to the *rpoC* gene.

Single colonies derived from this growth (24–96 hours on BHIS agar) were subjected to the asymmetric PCR-digestion assay to determine the orientation of the flagellar switch. Individual colonies were enriched for one orientation of the flagellar switch. All colonies tested yielded either 312 and 353 bp fragments indicating the switch in the published orientation (Fig 3A) or 418 and 247 bp fragments indicating the switch in the inverse orientation (Fig 3B). However, the asymmetric PCR-digestion has limited sensitivity for detection of low abundance targets. Thus, we used quantitative PCR (qPCR) of genomic DNA to quantify the frequency of bacteria with the flagellar switch in each orientation. In isolated colonies that showed fragments indicating the switch is in the published orientation by asymmetric PCR-digestion (Fig 3A), 89.5% (+/- 6.6% SD) of the population had the switch in that orientation by qPCR (Fig 3C). Similarly, in colonies with the switch predominantly in the inverse orientation by asymmetric PCR-digestion (Fig 3B), 96.3% (+/- 2.2% SD) of the population has the switch in that orientation by qPCR (Fig 3C). These data indicate that asymmetric PCR-digestion successfully identifies colonies that are enriched for the flagellar switch in a single orientation. Furthermore, the orientation of the flagellar switch remains stable during growth on an agar surface for at least 24 hours and switch orientation did not affect growth (S2 Fig).

We note that during these experiments, we observed two distinct colony morphologies based on colony texture and edge—a smooth, circular (SC) morphotype, and a rough, filamentous (RF) morphotype (S3 Fig). The percentage of RF colonies increased, and the percentage of SC colonies decreased over time (S3 Fig). Bacteria from SC and RF colonies maintained their respective characteristic morphologies after passaging (S3 Fig). Colony morphology was not attributable to the orientation of the flagellar switch, as 72% of bacteria in the SC colonies had the flagellar switch in the published orientation, and 64% of bacteria in the RF colonies had the flagellar switch in the inverse orientation (S3 Fig).

### The orientation of the flagellar switch controls downstream flagellar gene expression

**Fig 4:**
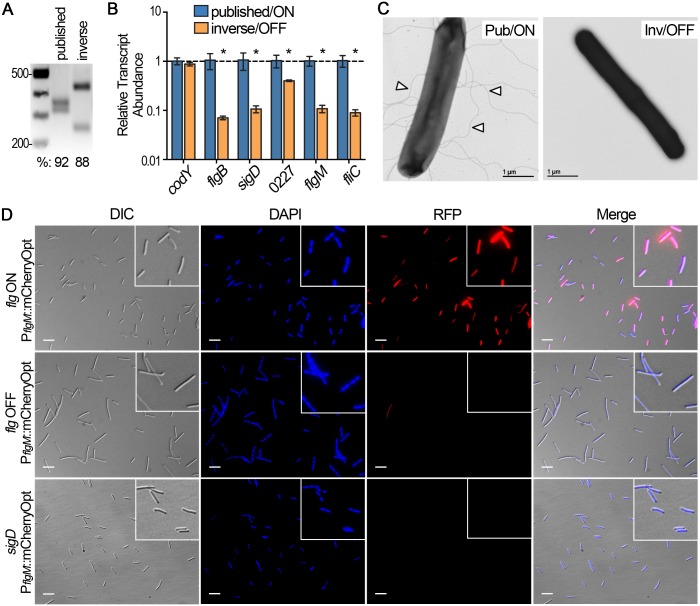
The orientation of the flagellar switch impacts the expression of the downstream flagellar genes. (A) Asymmetric PCR-digestion assay performed on *C*. *difficile* R20291 isolates with the flagellar switch in the published and inverse orientations respectively. (B) qRT-PCR was used to determine the abundance of representative flagellar gene transcripts in isolates with the flagellar switch in the published and inverse orientation. Four independent isolates were tested, and *Ct* values for each flagellar gene and the *codY* gene (non-regulated control) were normalized to those of the housekeeping gene *rpoC*; the published orientation samples were arbitrarily chosen as the reference condition. Shown are the means and standard deviations. * *p* < 0.05 by t-tests comparing mean transcript abundances between published and inverse samples, n = 4. (C) Visualization of flagella by transmission electron microscopy at 25,000X magnification. Size bars = 1 micron. Representative images of bacterial flagellar switch isolates are shown. Arrowheads indicate flagella. (D) Micrographs of enriched *flg* ON, *flg* OFF, and a *sigD* mutant transformed with the pP_*flgM*_::*mCherryOpt* reporter. Channels used are indicated for each column; the fourth column images are a merge of the DIC, DAPI, and RFP. RFP positive and negative bacteria were visually enumerated relative to the DIC and DAPI channels, and quantifications are shown in S4 Fig. White bars = 10 microns.

The enrichment of two populations based on the orientation of the flagellar switch allowed us to determine the impact of switch orientation on downstream gene expression. We purified colonies with the flagellar switch in the published and inverse orientations (Fig 4A) to assess gene expression. The abundances of early and late stage flagellar gene transcripts in the isolates were compared by quantitative reverse transcriptase PCR (qRT-PCR). Bacteria with the flagellar switch in the inverse orientation exhibited significantly reduced abundance of early stage flagellar gene transcripts, such as *flgB* and *sigD/fliA*, compared to bacteria with the flagellar switch in the published orientation (Fig 4B) [23,44]. Accordingly, the abundances of the σ^D^-dependent, late stage flagellar gene transcripts CDR20291_0227 (autolysin), *flgM* (flagellar anti-sigma factor), and *fliC* (flagellin) (Fig 4B) were also significantly decreased in bacteria with the flagellar switch in the inverse orientation. Consistent with this, transmission electron microscopy showed that the majority of bacteria from colonies with the flagellar switch in the published orientation displayed flagella (72% flagellated, n = 144), whereas bacteria from colonies with the switch in the inverse orientation were largely non-flagellated (98% non- flagellated, n = 163) (Fig 4C). We also note that *C*. *difficile* strain R20291 was previously reported to produce a single flagellum [31], but we observed peritrichous flagella on our R20291 isolate.

In addition, we generated a transcriptional fusion of *mCherryOpt*, encoding a Red Fluorescent Protein-derivative (RFP) optimized for translation in *C*. *difficile* [72,73], to the *flgM* promoter. The *flgM* gene was previously determined to be positively regulated by σ^D^ [23,44]. As σ^D^ is encoded in the *flgB* operon, it is indirectly phase variable and serves as an indicator of the status of the flagellar switch. This plasmid-borne reporter (P_*flgM*_::*mCherryOpt*) was introduced into *C*. *difficile* R20291 isolates with the switch in the published or inverse orientation. In the isolate with the switch in the published orientation, the majority of bacteria were mCherryOpt positive: 76.7% (+/- 9.8% SD). In the isolate with the switch in the inverse orientation, few bacteria were mCherryOpt positive: 2.4% (+/- 2.2% SD) (Fig 4D). These values are consistent with those obtained using qPCR to directly evaluate the switch orientation in these populations (Fig 3C). Differences in the frequency of the switch orientation in enriched *flg* ON populations could be due to additional flagellar gene regulators that function independently of the *flg* switch, such as c-di-GMP [59,62]. Negative controls bearing promoterless *mCherryOpt (*::*mCherryOpt*) lacked fluorescence (S4 Fig); positive controls with inducible *mCherryOpt* (P_*tet*_::*mCherryOpt*) showed more uniform red fluorescence (S4 Fig). We additionally constructed a *sigD* mutant in R20291 by Targetron insertional mutagenesis as a negative control (S5 Fig). The *sigD* mutant containing the P_*flgM*_::*mCherryOpt* reporter lacked mCherryOpt fluorescence, indicating that *flgM* promoter activity is dependent on σ^D^, and therefore on the flagellar switch (Fig 4D).

Taken together, these data indicate that the orientation of the flagellar switch controls flagellar gene expression and are consistent with phase variable gene expression. Thus, bacteria with the flagellar switch in the published orientation express flagellar genes and produce flagella and are thus flagellar phase ON (“*flg* ON” hereafter); bacteria with the switch in the inverse orientation show decreased flagellar gene expression and flagellum biosynthesis and are comparatively flagellar phase OFF (“*flg* OFF” hereafter).

### Motility medium spatially segregates flagellar phase variant populations

**Fig 5:**
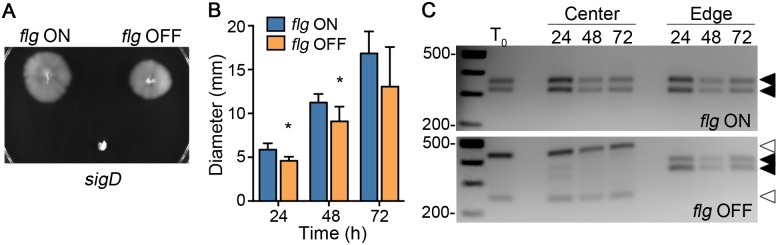
Motility medium spatially segregates flagellar phase variant populations. (A) The motility of *C*. *difficile* R20291 *flg* ON and OFF isolates was evaluated in soft agar medium. A non-motile R20291 *sigD* mutant was included as a control. (B) The motility of *flg* ON and OFF isolates was quantified by measuring the diameters every 24 hours for 3 days, and means and standard deviations are shown. * *p* < 0.05 by t-tests comparing values at each time point. The data are combined from two independent experiments with four biological replicates of each *flg* phase. (C) At 24, 48 and 72 hours, bacteria were sampled from the center and edges of the growth in the motility assays for *flg* ON and OFF isolates (top and bottom panels, respectively) and subjected to asymmetric PCR-digestion assays. Black arrows indicate products for the ON orientation; open arrows, the OFF orientation. Shown are representative images from 2 independent experiments, each with two biological replicates of each *flg* phase.

Given that *flg* OFF bacteria were deficient in flagellum biosynthesis (Fig 4), we predicted that *flg* OFF bacteria would be non-motile compared to motile *flg* ON bacteria. To test this, enriched *flg* ON and OFF isolates were examined for the ability to swim through BHIS-0.3% agar [59]. The R20291 *sigD* mutant was used as a non-motile control. The *flg* OFF bacteria appeared motile (Fig 5A), though they displayed a modest but statistically significant reduction in swimming diameter at 24 and 48 hours post-inoculation compared to *flg* ON isolates (Fig 5B).

We reasoned that the enriched *flg* OFF inoculum would result in spatial restriction of non-motile bacteria to the inoculation site. In contrast, the small fraction of *flg* ON bacteria (detectable by qPCR and fluorescent reporters but below the limitation of detection by asymmetric PCR digestion assay) would be capable of motility and chemotaxis and therefore expand from the inoculation site, spatially segregating from *flg* OFF bacteria. To test this, for *flg* ON and OFF isolates cultured in motility agar, we sampled bacteria from the inoculation site (center) and from the leading edge at 24, 48, and 72 hours and determined the orientation of the switch using the asymmetric PCR-digestion assay. As expected, *flg* ON maintained the flagellar switch in the ON orientation at all time points at both the center and edge (Fig 5C, top). In contrast, *flg* OFF bacteria from the center contained the switch primarily in the OFF configuration, but bacteria at the leading edge had the switch to the ON orientation by 24 hours (Fig 5C, bottom). The *flg* ON orientation was subsequently preserved along the leading edge at 48 and 72 hours (Fig 5C, bottom). These results indicate that growth in motility medium introduced a selection barrier that restricted *flg* OFF bacteria to the inoculation site and allowed the expansion of a low frequency population of *flg* ON bacteria.

### The orientation of the flagellar switch controls production of the glucosylating toxins

**Fig 6:**
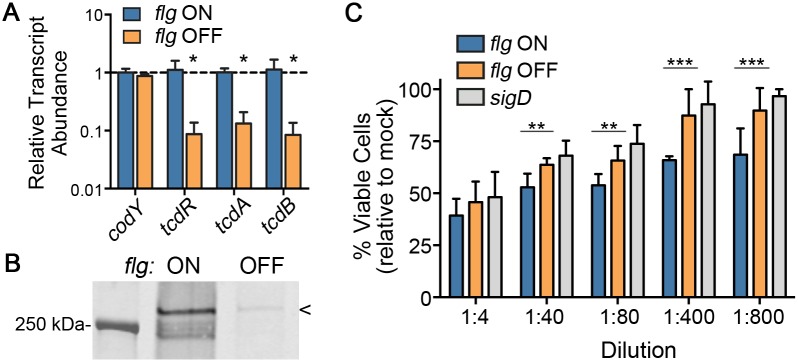
The orientation of the flagellar switch impacts toxin production. (A) qRT-PCR was used to determine the abundance of the indicated transcripts in *flg* ON and OFF isolates of *C*. *difficile* R20291. Four independent isolates were tested, and C*t* values for each gene were normalized to those of the housekeeping gene *rpoC*; the *flg* ON samples were arbitrarily chosen as the reference condition. Shown are means and standard deviations. * *p* < 0.05 by t-tests comparing mean transcript abundances between *flg* ON and OFF samples, n = 4. (B) TcdA protein levels in cell lysates of *flg* ON and OFF isolates were evaluated by western blot. Shown is a representative image for three independent experiments each with at least three replicates of each *flg* phase. (C) The *flg* ON and OFF isolates, as well as the *sigD* mutant control, were grown to stationary phase in TY medium. The supernatants were serially diluted and applied to Vero cells for 24 hours. Cell viability was assessed using the CellTiter Glo assay. Data are combined from two independent experiments each with four replicates of *sigD* mutant and *flg* phase variants, and means and standard deviations are shown. ** *p* < 0.01, *** *p* < 0.001 by one-way ANOVA comparing the means for each dilution.

The flagellar alternative sigma factor, σ^D^, controls transcription of genes within the Pathogenicity Locus (PaLoc) in addition to late stage flagellar genes in *C*. *difficile* [22,23,44]. Therefore, we predicted that the flagellar switch, which impacts the expression of *sigD* (Fig 4B), also indirectly controls the expression of the toxin genes *tcdA* and *tcdB* by activating the expression of the toxin sigma factor gene *tcdR*. We compared the expression of *tcdA*, *tcdB* and *tcdR* in *flg* ON and OFF isolates using qRT-PCR. The abundances of the *tcdR*, *tcdA* and *tcdB* transcripts were significantly reduced in *flg* OFF bacteria compared to *flg* ON (Fig 6A). Consistent with these results, TcdA protein level was also decreased in cell lysates of *flg* OFF bacteria compared to lysates of *flg* ON bacteria (Fig 6B). TcdB levels were below the limit of detection by western blot.

TcdA and TcdB are exported protein toxins that inactivate host cell Rho and Rac GTPases through glucosylation, resulting in actin depolymerization and reduced host cell viability [13–16]. To determine the effect of the flagellar switch on *C*. *difficile* cytotoxicity, filter-sterilized supernatants from *flg* ON and OFF isolates were tested in a cell viability assay with Vero cells. *Flg* ON isolates were significantly more cytotoxic compared to *flg* OFF isolates and a *sigD* control (Fig 6C). Taken together, these data demonstrate that the flagellar switch orientation controls toxin production in addition to flagellum biosynthesis and swimming motility, indicating that these major virulence factors are coordinately phase variable in *C*. *difficile*.

### The flagellar switch mediates regulation at the posttranscriptional level

Multiple mechanisms driving phase variable gene expression have been described: general homologous recombination, conservative site-specific recombination, short sequence repeat and slip strand mispairing, and DNA methylation [66,74,75]. Classically, an invertible DNA element that undergoes site-specific recombination contains a promoter as the regulatory feature, and the orientation of the invertible DNA element (and promoter) determines whether the downstream genes are expressed. For example, in *E*. *coli* the orientation of the *fimS* switch, which contains a promoter, controls type I fimbrial gene expression [76]. In contrast, in *C*. *difficile*, the genetic switch that regulates expression of *cwpV* operates after transcription initiation [68]. In *cwpV* phase ON bacteria, transcriptional read-through of the 5’ UTR and the *cwpV* coding sequence occurs. In *cwpV* phase OFF bacteria, the inversion of a DNA sequence in the 5’ UTR leads to formation of a Rho-independent transcriptional terminator and results in premature transcription termination preventing expression of *cwpV*.

**Fig 7:**
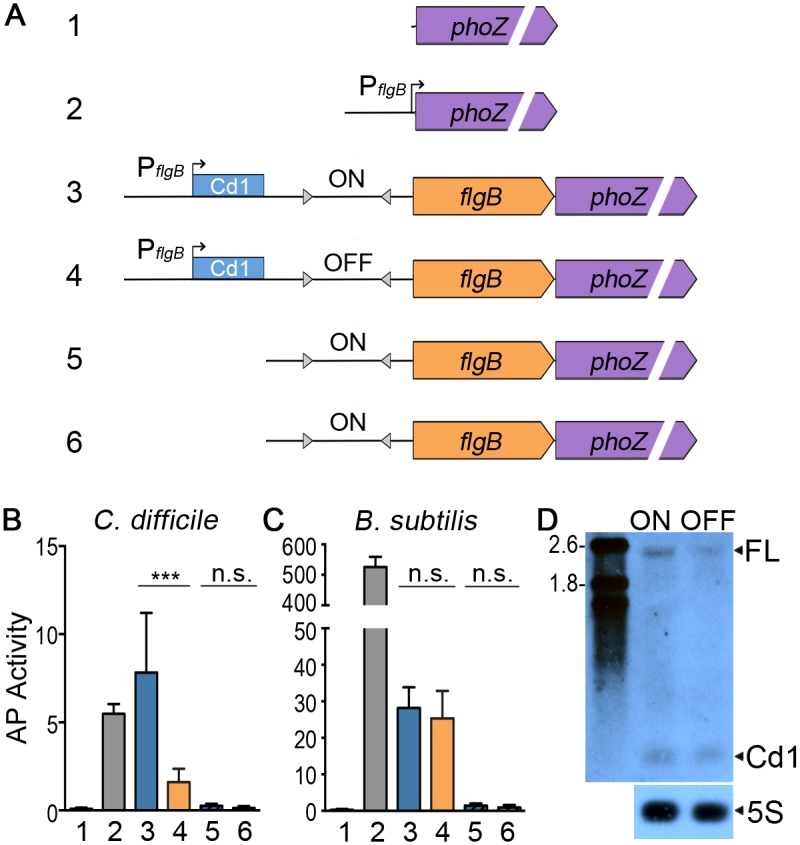
The orientation of the flagellar switch controls flagellar gene expression post-transcription initiation. (A) Diagram of the reporter gene fusions (S1 Methods). The known *flgB* operon promoter, the Cd1 c-di-GMP riboswitch, and the orientation of the flagellar switch are indicated, if present. (B,C) The fusions in A were integrated into the *C*. *difficile* R20291 chromosome (left) or the *B*. *subtilis* BS49 chromosome via Tn916 [112]. Alkaline phosphatase (AP) activity was measured as described previously [77]. Means and standard deviations are shown. *** *p* < 0.001 by one-way ANOVA and Bonferroni’s multiple comparisons test, n = 8. n.s. = not significant. (D) Northern blot detection of the *phoZ*-containing transcripts from *C*. *difficile* R20291 bearing fusions 3 or 4. The full length (FL) transcript of ~2400 nt is indicated. 5S RNA served as the loading control. The image is representative of three biological replicates for each strain.

To begin to define the mechanism by which the flagellar switch controls gene expression, we generated a series of alkaline phosphatase (AP) transcriptional reporters (Fig 7A) [77]. First, the *phoZ* reporter gene was placed downstream of *flgB* (first gene of the *flgB* operon), under the control of the native *flgB* promoter and the full 498 bp 5’ UTR with the flagellar switch (FS) in either the phase ON or OFF orientation (P_*flgB*_-5’UTR(FS^ON/OFF^)-*flgB*::*phoZ*, Fig 7A, #3 & 4). To determine if the flagellar switch contains a promoter, a truncated 307 bp 5’ UTR retaining the flagellar switch (either phase ON or OFF orientation) but lacking the Cd1 riboswitch and native *flgB* promoter was placed upstream of *flgB*::*phoZ* (FS^ON/OFF^-*flgB*::*phoZ*, Fig 7A, #5 & 6). A transcriptional fusion of *phoZ* to the *flgB* promoter (P_*flgB*_::*phoZ*, Fig 7A, #2) served as a positive control for the assay, and a promoterless construct (::*phoZ*, Fig 7A, #1) was included as a negative control. All reporter fusions were integrated onto the chromosome of an R20291 *flg* ON isolate, and AP activity was assessed. In parallel with every AP assay, an asymmetric PCR-digestion assay was performed to ensure that the flagellar switches were in the expected orientation for both the native locus and the reporter fusion (S6 Fig). As anticipated, the promoterless control showed no AP activity, and the presence of the *flgB* promoter significantly increased activity (Fig 7B). The P_*flgB*_-5’UTR(FS^OFF^)-*flgB*::*phoZ* reporter strain had significantly reduced activity compared to the P_*flgB*_-5’UTR(FS^ON^)-*flgB*::*phoZ* reporter strain (Fig 7B), consistent with previous results (Fig 4). No AP activity was detected in strains bearing the FS^ON/OFF^-*flgB*::*phoZ* fusions (Fig 7B). Similar results were obtained using the same six reporter fusions integrated onto the chromosome of a R20291 with the native flagellar switch in the OFF orientation, although AP activity was lower overall (S7 Fig). These results suggest that the flagellar switch does not contain a promoter to initiate transcription independently of the *flgB* promoter.

We considered alternative mechanisms by which expression could be inhibited in bacteria with the switch in the OFF orientation: (1) the formation of a Rho-independent transcriptional terminator, (2) the requirement for a *trans*-acting element such as an RNA binding protein or small non-coding RNA that selectively inhibits transcription, or (3) a Rho-dependent terminator. To examine these possibilities, we evaluated the activity of the *phoZ* reporters in a heterologous bacterium, *B*. *subtilis*, in which *C*. *difficile*-specific factors will be absent, but features inherent to the flagellar switch will be preserved. The reporter fusions (Fig 7A) were integrated into the *B*. *subtilis* BS49 chromosome, and the orientation of the flagellar switch was monitored by orientation-specific PCR (S6 Fig). As in *C*. *difficile*, the positive control strain with the P_*flgB*_::*phoZ* fusion showed high AP activity, but no activity was observed for the::*phoZ* negative control (Fig 7B). Also consistent with *C*. *difficile*, *B*. *subtilis* with the FS^ON/OFF^-*flgB*::*phoZ* fusions produced negligible AP activity, indicating the absence of a promoter in the flagellar switch (Fig 7C). In contrast to the *C*. *difficile* results, we observed comparable AP activity in *B*. *subtilis* with P_*flgB*_-5’UTR(FS^ON/OFF^)-*flgB*::*phoZ* reporters (Fig 7C). Thus, reduced gene expression as a result of the flagellar switch in the OFF orientation is specific to *C*. *difficile*, indicating that the regulatory feature is not inherent to the sequence, instead supporting the role of a *trans*-acting factor that is specific to *C*. *difficile*.

When considered along with the qRT-PCR data from Figs 4B and 6A, these results suggest regulation post-transcription initiation from the *flgB* operon promoter and implicates changes in RNA stability or premature termination. Therefore, we used northern blot analysis to determine if transcription terminates prematurely or if the transcript is destabilized when the flagellar switch is in the OFF orientation. We were unable to detect the full-length transcript for the *flgB* operon (~ 23 kb) using a probe specific to the Cd1 region of the 5’ UTR, so we used strains with the P_*flgB*_-5’UTR(FS^ON/OFF^)-*flgB*::*phoZ* reporters in the R20291 *flg* ON background to evaluate premature transcription termination. We observed an RNA corresponding to a full-length transcript of ~2400 nt for the P_*flgB*_-5’UTR(FS^ON^)-*flgB*::*phoZ* reporter but reduced transcript for the P_*flgB*_-5’UTR(FS^OFF^)-*flgB*::*phoZ* reporter (Fig 7D). Furthermore, we did not detect a smaller transcript species other than termination through the Cd1 riboswitch, which eliminates the possibility of Rho-dependent or -independent termination unless the transcript is degraded faster than we can detect by northern blot. Collectively, the reporter assays in *C*. *difficile flg* ON and OFF and in *B*. *subtilis* with northern blot analysis suggest that regulation via the flagellar switch occurs post-transcription initiation and involves an unidentified *trans*-acting element that destabilizes the mRNA to reduce gene expression in *flg* OFF bacteria.

### RecV, a tyrosine recombinase, catalyzes recombination at the flagellar switch in both orientations

**Fig 8:**
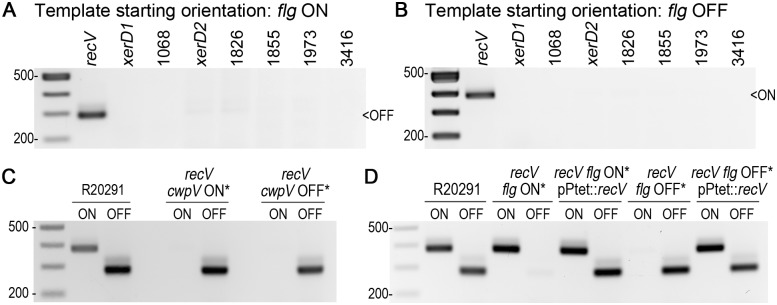
Identification of the recombinase that mediates inversion of the flagellar switch. (A,B) Orientation-specific PCR (Fig 1B) assays to identify the conserved recombinase that catalyzes inversion at the flagellar switch from ON to OFF (A) and OFF to ON (B). The gene name or R20291 locus tag numbers for the eight conserved recombinases are shown. A 375 bp product indicates the ON orientation; a 281 bp product indicates the OFF orientation. (C) Orientation-specific PCR for the flagellar switch to determine whether the *recV cwpV* locked ON and OFF mutants were locked for the flagellar switch. (D) Orientation-specific PCR for the flagellar switch in complemented *recV* mutants (pP_*tet*_::*recV*) and controls.

Serine and tyrosine recombinases catalyze site-specific recombination to mediate DNA inversion at phase variable genetic switches in a RecA-independent manner [78,79]. In *E*. *coli*, two recombinases catalyze inversion at the fimbrial switch; FimB can catalyze both orientations, whereas FimE is restricted to ON to OFF inversion events [80,81]. A conserved tyrosine recombinase called RecV catalyzes strand exchange in both orientations at the *cwpV* switch in *C*. *difficile* [68,82–84]. We postulated that the recombinase(s) which catalyze inversion at the *flg* switch would be present in all published *C*. *difficile* genomes with intact flagellum biosynthesis genes. We identified eight conserved serine or tyrosine recombinases, including RecV (CDR20291_1004), and used a two-plasmid system in a heterologous bacterium to identify the flagellar switch recombinase(s) [68]. One plasmid contains one of the eight recombinase genes cloned downstream of an anhydrotetracycline (ATc) inducible promoter [85], and the second plasmid contains one of the P_*flgB*_-5’UTR(FS^ON/OFF^)-*flgB*::*phoZ* reporters (Fig 7A, #3 & 4). The plasmids were transformed into *E*. *coli*, induced with ATc, and orientation-specific PCR was used on purified plasmids to determine if inversion occurred. We found that RecV was sufficient to catalyze recombination of the flagellar switch from the ON to OFF (Fig 8A) and OFF to ON (Fig 8B) orientations in *E*. *coli*; the other seven were unable to promote recombination. These data indicate that the RecV recombinase is sufficient to catalyze inversion in both orientations at both the flagellar and *cwpV* switches in a heterologous organism. These data do not rule out the possibility of an additional recombinase that requires a *trans*-acting element, such as a recombination directionality factor [86], to catalyze inversion at the flagellar switch.

To determine whether RecV is involved in inversion of the flagellar switch in *C*. *difficile*, we first overexpressed *recV* in the backgrounds of R20291 *flg* ON and OFF using the anhydrotetracycline (ATc)-inducible expression vector to determine whether RecV would promote inversion to the opposite orientation [87]. We found that overexpression of *recV* in the *flg* ON and OFF backgrounds leads to a mixed population of both orientations compared to empty vector, regardless of the presence of ATc (S8 Fig, panel A). Transcription of *recV* in the ATc-inducible expression vector was leaky in the absence of inducer (S8 Fig, panel B), which is in line with previous work [87,88].

### RecV mutants are phase-locked for flagellum and toxin production

The *C*. *difficile* R20291 *recV* mutants were previously shown to be phase-locked for CwpV production [83,84]. The identification of RecV as the recombinase mediating flagellar switch inversion suggests that mutation of *recV* would similarly result in phase-locked phenotypes with respect to flagellum and toxin production. To evaluate the requirement of RecV for flagellar phase variation in *C*. *difficile*, we obtained two *C*. *difficile* R20291 mutants with ClosTron insertions in *recV* (kind gift from Dr. Louis-Charles Fortier). One mutant contains the *cwpV* switch locked in the ON orientation, and the other, the OFF orientation [82,83]. Both mutants contain the flagellar switch in the OFF orientation (herein, “*flg* OFF*”), as determined using orientation-specific PCR (Figs 1B and 8C).

To obtain a *recV* mutant in which the flagellar switch is locked in the ON orientation, *recV* was expressed from a plasmid to allow inversion of the flagellar switch (Fig 8C). This complemented strain was then passaged in the absence of antibiotic selection to allow loss of the plasmid. Thiamphenicol-sensitive clones were screened for flagellar switch orientation to identify *recV* mutants that are *flg* ON (herein, “*flg* ON*”) (S9 Fig).

**Fig 9:**
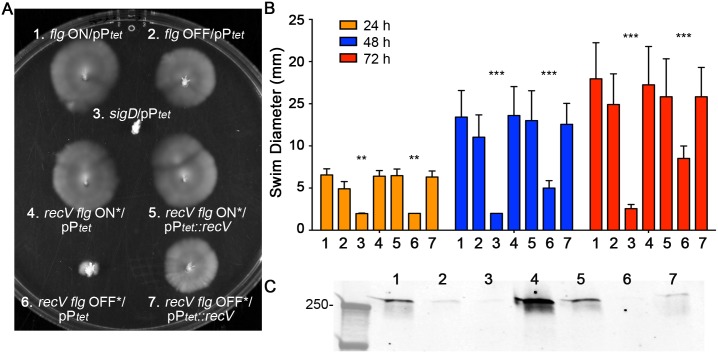
Mutation of *recV* results in phase-locked motility and toxin production. (A,B) *C*. *difficile* strains were assayed for motility in BHIS-0.3% agar. (A) Photographs were taken every 24 hours, and a representative image from 48 hours is shown. (B) Measurements of the diameters of motility were taken at 24, 48 and 72 hours. Shown are means and standard deviations from six biological replicates. ** p < 0.01, *** p < 0.001, by two-way ANOVA and Tukey’s multiple comparison test, comparing to *flg* ON/pP_*tet*_. (C) TcdA protein levels in cell lysates were evaluated by western blot. Shown is a representative image of three independent assays. (B,C) Numbers indicate the strain, as indicated in panel A.

The *recV flg* ON* and OFF* mutants, each bearing vector or *recV* under the control of the P_*tet*_ ATc-inducible promoter, were assayed for swimming motility in BHIS-0.3% agar. A non-motile *sigD* mutant and enriched *flg* ON and OFF isolates were included as controls. Unlike the enriched *flg* OFF isolate that appeared motile due to a small frequency of *flg* ON bacteria, the *recV flg* OFF* mutant was non-motile at 24 hours incubation (Fig 9A and 9B). This phase-locked phenotype was dependent on RecV, as complementation with *recV in trans* restored motility. In contrast, the *recV flg* ON* mutant showed motility, and providing *recV in trans* had no measurable effect in this assay. We note that extended incubation (greater than 24 hours) of the *recV flg* OFF* mutant results in motile progeny (S10 Fig). These motile bacteria retain the flagellar switch in the OFF orientation based on orientation-specific PCR and DNA sequencing results (S10 Fig), so the motile phenotype is due to suppressor mutations.

We next evaluated toxin production by the *recV flg* ON* and OFF* mutants by measuring TcdA production by western blot. As shown above, the enriched *flg* OFF isolate produced less TcdA than the enriched *flg* ON isolate (Fig 9C, lanes 1,2). TcdA levels were greater in the *recV flg* ON* mutant compared to the enriched *flg* ON isolate. The difference is likely attributable to the presence of ~10.5% or 23.2% of bacteria with the flagellar switch in the OFF orientation remaining in the enriched *flg* ON isolate based on qPCR and fluorescence microscopy estimates, respectively (Figs 3C and 4D). TcdA abundance in the *recV flg* OFF* mutant was comparable to that in the *sigD* mutant, which was below the limit of detection. Provision of *recV in trans* in either the *recV flg* ON* or OFF* mutant, which results in heterogeneity in the orientation of the flagellar switch (Fig 8D), resulted in intermediate TcdA levels (Fig 9C, lanes 5,7). The observed differences in TcdA levels among the strains appear to depend on the proportion of *flg* ON and OFF bacteria in the population. These results indicate that RecV mediates flagellum and toxin phase variation, in addition to CwpV phase variation, in *C*. *difficile*.

## Discussion

Colonization is a prerequisite step to diarrheal disease development in *C*. *difficile* infection. The identification of colonization factors and the mechanisms controlling their production is an active area of study. Transcriptional regulators [51–53], a peptide-based quorum sensing system [29], and an RNA chaperone [54] activate or repress flagellar gene expression, albeit the exact regulatory mechanisms remain undetermined. Prior to this study, Cd1, a class I c-di-GMP sensing riboswitch, was the only identified *cis*-acting regulatory element to control flagellar gene expression [59,61,62]. Here, we report an additional *cis*-acting regulatory element, a “flagellar switch,” present upstream of the early stage flagellar operon.

The flagellar switch is the fourth identified *C*. *difficile* inversion site (Cdi4) and consists of a 154 bp sequence flanked by 21 bp imperfect inverted repeats [69]. The flagellar switch is functional, undergoing site-specific recombination in at least two different *C*. *difficile* ribotypes, 027 and 017. The flagellar switch inverted repeats are conserved in all sequenced *C*. *difficile* genomes that contain the flagellum biosynthesis genes, except the 630 genome has shortened 20 bp inverted repeats (S11 Fig). The flagellar switch appears locked ON in *C*. *difficile* 630, which may be due to the shortened inverted repeats. Alternatively, the 630 genome (012 ribotype) may differ in RecV transcription, production, or activity compared to the 027 and 017 ribotype strains, although there are no obvious differences in the *recV* promoter or open reading frame sequences to indicate that this is the case. Genetic evidence suggests that reduced inverted repeat length can impair recombination at the *cwpV* switch in *C*. *difficile* [68]. The *cwpV* switch (Cdi1) controls the production of a large cell wall protein that promotes bacterial aggregation *in vitro* and is postulated to promote intestinal colonization [68,82]. CwpV may also contribute to *C*. *difficile* survival in the host by conferring resistance to predation by bacteriophages [84]. Deposition of CwpV on the surface of *C*. *difficile* reduces phage adsorption and specifically prevents the injection of phage DNA into the bacterial cell [84]. The other two identified inversion sites in *C*. *difficile*, although not yet demonstrated to be functional switches, are under active investigation.

Unlike the *cwpV* switch that controls the expression of a single structural gene, the flagellar switch controls the expression of both structural and regulatory genes, including *sigD*. Flagellar phase ON bacteria, as well as a *recV* mutant with the flagellar switch locked in the ON orientation, display peritrichous flagella, engage in swimming motility, and produce the glucosylating toxins. Flagellar phase OFF bacteria and *recV* mutants with the flagellar switch locked in the OFF orientation are attenuated for transcription of flagellar genes, and therefore grow as aflagellate, non-motile bacteria also attenuated for toxin production. Thus, the flagellar switch coordinately controls the production of a colonization factor and essential virulence determinants by impacting *sigD* expression. Moreover, the flagellar switch orientation is expected to affect additional genes in the σ^D^ regulon, such as metabolic pathways, multiple cell wall proteins, metabolic transporters for amino acids and divalent cations, and several transcriptional regulators [23].

Classically, genetic switches that toggle between two orientations and facilitate phase variable expression of downstream genes contain a promoter to alter transcription initiation [66]. Transcriptional reporter data excluded the presence of a promoter within the flagellar switch, indicating that the switch controls downstream gene expression after transcription initiation from the *flgB* operon promoter has occurred. Indeed, the switch is within the previously defined 5’ UTR of the *flgB* (F3) operon, downstream of the c-di-GMP riboswitch [62]. This arrangement of regulatory elements suggests that flagellar phase variation is only relevant when c-di-GMP levels are sufficiently low to permit transcriptional read-through beyond the riboswitch and into the flagellar switch. Regulation post-transcription initiation by genetic switches has been described previously. The *cwpV* switch in *C*. *difficile* also lacks promoter activity and instead regulates *cwpV* expression post-transcriptionally [68]. When the switch is in the *cwpV* phase OFF orientation, the mRNA adopts a structure containing a Rho-independent terminator resulting in premature transcription termination [68]. In *E*. *coli*, the *fimE* mRNA is subject to regulation post transcription initiation. FimE is encoded immediately 5’ of *fimS*, and when *fimS* is in the OFF orientation, the *fimE* mRNA forms a Rho-dependent terminator that reduces transcript stability [89]. If the flagellar switch in *C*. *difficile* contained a Rho-independent terminator, we would expect no AP activity in *B*. *subtilis* when the flagellar switch was in the OFF orientation. However, AP activity was indistinguishable between the *flg* ON and OFF reporters in *B*. *subtilis*, suggesting a *trans*-acting regulatory element that is expressed only in *C*. *difficile*. We note that AP activity from the reporters was substantially higher in *B*. *subtilis* compared to *C*. *difficile*; it is possible that a regulatory factor in *B*. *subtilis* masked the unidentified *trans*-acting regulatory factor. Moreover, northern blot analysis suggests a mechanism independent of both Rho-dependent and independent termination since we were unable to detect a truncated transcript from the phase OFF condition. Collectively, our results suggest that the early stage flagellar operon mRNA is destabilized or degraded when the flagellar switch DNA is in the OFF orientation. We speculate that a constitutively-expressed *C*. *difficile*-specific regulatory factor, either an RNA binding protein or small, non-coding RNA, binds to the leader sequence of the mRNA to directly or indirectly degrade or destabilize the mRNA. Thus, the flagellar switch in *C*. *difficile* represents a novel system that requires an unknown additional regulatory factor to function as an ON/OFF switch.

In many mucosal pathogens, phase variation is a regulatory feature that modulates the expression of an immunostimulatory cell surface structure [66]. Flagellar phase variation occurs in *Clostridium chauvoei* [90], *Salmonella enterica* serovar *enterica* Typhimurium [91,92], *Helicobacter pylori* [93], and *Campylobacter jejuni* [94]. Wild strains of *B*. *subtilis* grown under planktonic conditions bifurcate into two distinct populations: flagellated, motile single bacteria and aflagellate, non-motile chains of bacteria [95]. Expression and activity of σ^D^ determines whether these populations arise in *B*. *subtilis*. Expression of *swrA*, which encodes a master regulator of swarming motility, is subject to phase variation by slip strand mispairing in the coding sequence [95–97]. SwrA controls transcription of the flagellar and chemotaxis (*fla/che*) operon by regulating the activity of the transcriptional regulator DegU [98]. SigD, encoded within the *fla/che* operon, also positively regulates *swrA* transcription [98]. Thus, a feedforward loop resulting from *swrA* expression introduces a bias for σ^D^ ON bacteria that are flagellated and motile. These and other data present a model in which flagellar gene expression in *B*. *subtilis* is bistable [97]. Although additional work is needed to determine if *C*. *difficile* flagellar gene expression meets the criteria of bistability [99–101], inversion of the *C*. *difficile* flagellar switch similarly results in phenotypic heterogeneity that would confer an advantage in an environment that selects for one phenotype over the other [99–101]. Thus, *B*. *subtilis* and *C*. *difficile* may use distinct mechanisms to accomplish the same outcome.

We identified RecV as necessary and sufficient for inversion of the flagellar switch in both orientations, by over-expression of *recV* in *C*. *difficile* and in *E*. *coli* bearing the flagellar switch sequence, and by mutation of *recV* in *C*. *difficile* R20291. Notably, RecV was previously reported to control inversion of the *cwpV* switch in *C*. *difficile* [68,82]. The use of a single recombinase to control inversion of multiple genetic switches has been described previously. In *Bacteroides fragilis*, the Mpi recombinase controls inversion of at least 13 genetic switches, including seven switches that control expression of different capsular polysaccharide loci [102]. These switches are scattered through the genome and contain inverted repeats that harbor a 10 bp core consensus sequence [102]. In contrast, the inverted repeats and the flanking half sites for the *cwpV* and flagellar switches lack recognizable sequence identity, although the identification of additional RecV-controlled genetic switches might reveal a core consensus sequence. RecV may bind the recognition sequences with different affinities, allowing hierarchical inversion of switches bearing disparate inverted repeats. Regulation of *recV* transcription and/or RecV activity could therefore differentially affect inversion of the *cwpV* and flagellar switches. Hierarchical binding could be achieved solely by RecV, or via additional proteins that direct RecV to specific target sequences. The need for these binding partners may have been bypassed in *E*. *coli* due to over-expression of *recV*. It is also possible that *trans*-acting DNA binding proteins, such as a histone-like protein or recombination directionality factor (RDF) [86,103], influence DNA bending to affect the recombination reaction at RecV-controlled genetic switches. Such DNA binding proteins can integrate environmental signals to bias switch orientation and generate genetic variants that have a fitness advantage in that environment. For example, leucine-responsive regulatory protein and H-NS affect the recombination reaction at *fimS* in *E*. *coli* [104,105]. The existence of factors influencing RecV activity in *C*. *difficile* is supported by the observation that *C*. *difficile* R20291 carrying the prophage ϕCD38-2 has the *cwpV* switch biased to the ON orientation; *recV* transcription is unaltered [83]. Prophage infection of a *recV* mutant with the *cwpV* switch in the OFF orientation remains locked, suggesting that the phage does not encode a recombinase to promote inversion, but that instead a phage gene product modulates RecV activity [83]. ϕCD38-2 did not affect flagellum or toxin gene transcription in R20291, but *C*. *difficile* may produce another factor that similarly influences recombination within the flagellar (and *cwpV*) switch. Recent evidence demonstrates a role for RDFs in *C*. *difficile* physiology: an RDF pairs with a serine recombinase to excise a prophage-like element from a sporulation-specific sigma factor gene [106]. Future studies will explore how RecV can control multiple genetic switches with divergent inverted repeat sequences.

Ultimately, iterative combinations of *cwpV* and *flg* ON and OFF phenotypes may influence *C*. *difficile* fitness in the host intestinal environment. A dual *cwpV* and *flg* ON phenotype could facilitate penetration of the bacteriophage-rich colonic mucus [107], and/or increase bacterial attachment to and colonization of the intestinal mucosa. Conversion of one or both phenotypes to phase OFF might reduce the probability of the host simultaneously developing an antibody response to these immunostimulatory surface structures. The role of flagellum and toxin phase variation in *C*. *difficile* pathogenesis remains to be determined, though it stands to reason that both the phase ON and OFF phenotypes confer advantages during the course of an infection. The *flg* ON bacteria would be competent for efficient intestinal colonization for R20291 [31]. Yet purified flagellin from multiple *C*. *difficile* strains can activate host Toll-like receptor 5 (TLR5) and stimulate p38 MAPK activation and IL-8 secretion *in vitro* [47,108,109]. Although TLR5 is dispensable in a mouse model of CDI [49], recombinant *C*. *difficile* FliC is immunogenic and protective in a mouse model of CDI [110]. In addition, the *C*. *difficile* glucosylating toxins have been implicated in activation of the NLRP3 and Pyrin inflammasomes, which could promote pathogen clearance [48,111]. *C*. *difficile flg* OFF bacteria could thus evade TLR5 recognition and inflammasome stimulation, enhancing bacterial colonization and persistence within the host. Future studies will determine the contribution of flagellum and toxin phase variation to *C*. *difficile* virulence.

## Materials and methods

### Growth and maintenance of bacterial strains

The strains and plasmids used in this study are listed in S1 Table, and details on their construction are in S1 Methods. *C*. *difficile* was maintained in an anaerobic chamber (Coy Laboratories) at an atmosphere of 90% N_2_, 5% CO_2_, and 5% H_2_ and grown in Brain Heart Infusion medium (Becton Dickinson) supplemented with 5% yeast extract (Becton Dickinson) (BHIS) at 37°C. Bacteria were also cultured in Tryptone Yeast (TY) broth media where indicated. All *C*. *difficile* broth cultures were grown statically. *Escherichia coli* DH5α, BL21, and HB101(pRK24) were cultured at 37°C in Luria-Bertani (LB) medium with the indicated antibiotics for plasmid selection. *Bacillus subtilis* strain BS49 was grown in BHIS with the appropriate antibiotics. Antibiotics were used at the following concentrations: chloramphenicol, 10 μg/ml; thiamphenicol, 10 μg/ml; kanamycin, 100 μg/ml; ampicillin, 100 μg/ml; erythromycin, 5.0 μg/ml, lincomycin 20.0 μg/ml.

### Orientation-specific PCR assay

Spores of *C*. *difficile* strains 630*Δerm*, R20291, and ATCC 43598 were plated on BHIS supplemented with 0.1% sodium taurocholate (Sigma) (BHIS+TA). After 24 hours, individual colonies were grown in BHIS at 37°C. Overnight cultures were diluted 1:50 in fresh BHIS and grown to early stationary phase. An aliquot of each culture was diluted 1:5 into 10 mM Tris-HCl, pH 7.5, 1 mM EDTA (TE) buffer. Boiled lysates served as templates in PCR using primers designed for each strain based the genome sequences for strains 630 (Accession No. AM180355.1) and R20291 (Accession No. FN545816.1). The ribotype 017 strain ATCC 43598 has not been sequenced, so we used M68 (Accession No. FN688375.1) as a representative ribotype 017 strain to design primers for the flagellar switch. Primer sequences are listed in S2 Table. Primers R1614 (published/ON orientation) and R1615 (inverse/OFF orientation) were used for R20291. Primers R1622 (published/ON orientation) and R1623 (inverse/OFF orientation) were used for ATCC 43598. Primers R1751 (published/ON orientation) and R1752 (inverse/OFF orientation) were used for 630*Δerm*. All reactions used R857 as the reverse primer given the sequence identity between strains for the first gene in the early flagellar operon, *flgB*. The results shown are representative of three independent experiments each with at least two biological replicates for each strain.

### Asymmetric PCR-digestion assay

To amplify the flagellar switch, chromosomal DNA purified from *C*. *difficile* R20291, as previously described [112], served as the template in PCRs with primers R591 and R857, yielding a product of 665bp. The PCR products (500 ng) were digested with SwaI (NEB), which results in different products depending on the orientation of the flagellar switch: 312 bp and 353 bp for the published orientation, and 418 bp and 247 bp for the inverse orientation (Fig 2). A mixture of bacteria with the flagellar switch in the published and inverse orientation results in four bands at varying intensities. SwaI reaction products were separated in 2.5% agarose gels, which were stained with ethidium bromide (EtBr) for imaging with a G:BOX Chemi Imaging system. For all experiments with purified phase variant populations, three or four biological replicates of R20291 *flg* ON and OFF were assessed by the asymmetric PCR-digest assay to ensure a homogeneous population. We used ImageJ to perform a densitometry analysis of the digested bands to determine the relative proportions of bacteria with flagellar switch in the published/ON (312, 353 bp) and inverse/OFF (418, 247 bp) orientations. The pixel intensities of published/ON or inverse/OFF bands were divided by the total pixel intensity for that sample. These values were normalized to a standard curve generated by mixing known quantities of *flg* ON and OFF plasmid template and subjecting them to the asymmetric PCR-digestion assay, giving the percentage of bacteria in a sample with the switch in a given orientation.

To evaluate the effect of surface growth on the flagellar switch, *C*. *difficile* R20291 spores were plated on BHIS-TA to induce germination. After 24 hours, individual colonies were suspended and spotted onto four BHIS plates. Every 24 hours for four days, one plate was used for recovery of bacteria from colony for chromosomal DNA extraction. Once chromosomal DNA was collected, the orientation of the flagellar switch was determined using the asymmetric PCR-digestion assay. Densitometry analysis was performed using ImageJ software as described above. Values for bands corresponding to the published/ON and inverse/OFF orientations were compared to a standard curve. The data were combined from three independent experiments, each with two to four biological replicates.

### Purification of flagellar phase variant populations

*C*. *difficile* R20291 spores were plated on BHIS+TA. After 24 hours, six to ten individual colonies were suspended in BHIS, spotted onto individual BHIS plates, and grown 24–96 hours as indicated. Single colonies were derived from this growth (24–96 hours) by passaging onto BHIS plates and individually screened for the flagellar switch orientation by the asymmetric PCR digestion assay. A higher frequency of colonies with the flagellar switch in the OFF orientation was observed at later time points (96 hours). For all experiments using enriched flagellar phase variant populations, except the *phoZ* reporters, mCherry reporters, and cell viability assay, glycerol stocks were *not* made to ensure robust reproducibility of the phenotypes between independent experiments and reproducibility of the enrichment protocol. At least two to four biological replicates of *flg* phase ON and OFF populations were used for all experiments.

### Quantitative PCR analysis of the flagellar switch orientation

In all reactions, SYBR Green Real-Time qPCR reagents (Thermo Fisher) were used, with primers at a final concentration of 500 nM and an annealing temperature of 55°C. Titrated amounts of genomic DNA from *recV flg* ON* and *flg* OFF* were used to determine the amount of template necessary for the reactions and the primer efficiency. Primers for detection of the ON orientation (R2175 and R2177) had a PCR efficiency of 88.5% for the *recV flg* ON* DNA, and the primers for detection of the OFF orientation (R2176 and R2177) had a PCR efficiency of 84.5% for the *recV flg* OFF* DNA. To determine the frequency of the *flg* ON and OFF orientations in populations of enriched *C*. *difficile* R20291 *flg* ON and OFF isolates, three biological replicates of each were grown in BHIS medium to an OD_600_ of ~1.0, and chromosomal DNA was extracted as previously described [112]. Quantitative PCR was done using 4 ng of DNA from *flg* ON and OFF isolates. DNA copy number was calculated using the *ΔΔCt* method, with the *rpoC* gene as the indicated reference gene for DNA copy number.

### RNA extraction, cDNA synthesis, and quantitative reverse transcriptase PCR

Isolated *C*. *difficile flg* ON and OFF variants were grown overnight in BHIS medium, 1:50 in 3mL of BHIS medium and grown to stationary phase (OD_600_ of 1.8–2.0). RNA was isolated as described previously [59,112]. Briefly, cells were collected by centrifugation and lysed by bead beating in cold TriSURE (Bioline). Nucleic acid was extracted with chloroform (Sigma), precipitated from the aqueous phase with isopropanol, washed with ethanol, and suspended in RNase-free water. To remove contaminating genomic DNA, RNA was treated with TURBO DNase (Thermo Fisher) according to the manufacturer’s protocol. Synthesis of cDNA was done using a Tetro cDNA Synthesis kit (Bioline) and random hexamers according to the manufacturer’s instructions and including a no-reverse transcriptase control. Real-time PCRs were done using 2 ng of cDNA and SYBR Green Real-Time qPCR reagents (Thermo Fisher). Transcript abundance was calculated using the ΔΔCt method, with *rpoC* as the control gene and the indicated reference condition/strain [59].

### Swimming motility assay

*C*. *difficile* R20291, *flg* ON and OFF isolates, and *recV* mutants were assayed for flagellar motility as previously described [59]. An R20291 *sigD* mutant was included as a non-motile control in all experiments. Details regarding the generation of the *sigD* mutant are in S1 Methods and S5 Fig. Autoclaved 0.5 X BHIS with 0.3% agar (30 mL) was poured into 100 mm Petri dishes allowed to solidify overnight. These soft agar plates were kept in the anaerobic chamber for at least 4 hours prior to the experiment. Bacteria were grown in BHIS medium overnight, diluted 1:50 in fresh BHIS broth the next day, and grown to an OD_600_ of 1.0. Two microliters of *flg* ON, *flg* OFF, and *sigD* were inoculated into the agar, then incubated at 37°C. The diameter of growth was measured after 24, 48, and 72 hours; two perpendicular measurements were made for each swim site and averaged for each replicate. Three to four biological replicates were evaluated, each in technical duplicate (values averaged), in two independent experiments. Images were taken using the G:BOX Chemi imaging system with the Upper White Light illuminator. A Student’s t-test was used to determine statistical significance.

### Alkaline phosphatase assay

*C*. *difficile phoZ* reporter strains were grown from glycerol stocks on BHIS plates and incubated at 37°C. After 24 hours, 2–3 colonies of each reporter strains were grown overnight in TY medium and diluted 1:50 into BHIS medium. Late exponential phase cells (OD_600_ ≈ 1.5, 1.5 mL) were collected by centrifugation, the supernatant was discarded, and pellets were stored at -20°C overnight. Frozen pellets were thawed on ice and the alkaline phosphatase (AP) assay was performed as previously described [77]. *Bacillus subtilis* BS49 *phoZ* reporter strains were grown from glycerol stocks on BHIS-Erm plates under aerobic conditions, and the assay was done as above. Construction of AP reporters is described in S1 Methods.

### Detection of RNA by northern blot

*C*. *difficile phoZ* reporter strains were grown in 4mL of BHIS medium to OD_600_ 1.5. Bacteria were collected by centrifugation, and RNA was extracted as described above with the following exceptions. Four rounds of bead beating were done, and the purified RNA was rigorously treated with DNase I to ensure removal of contaminating DNA. Digoxigenin (DIG)-labeled DNA probes for the northern blot were generated by PCR using genomic DNA from R20291 as template for the Cd1 probe and a 5S rRNA (CDR20291_ r03) probe, a DIG High Prime kit was used (Roche) (S1 Table). Reagents from the NorthernMax-Gly Kit (Thermo Fisher) were used for electrophoresis and hybridization. RNA samples (15ug for mRNA probe and 500ng for rRNA probe) were briefly thawed on ice, mixed 1:1 with Glyoxal Load Dye, incubated at 50°C for 30 minutes, and electrophoresed in a 1% agarose gel made with 1X Gel Prep/Running Buffer. The agarose gel was imaged using the G:BOX Chemi Imaging system to confirm RNA integrity. The gel was briefly soaked in 20X SSC (3M NaCl and 300mM sodium citrate), then RNA was transferred via capillary action onto a nylon membrane (Amersham Hybond-N+) overnight in 20X SSC for 16 hours. RNA was crosslinked to the membrane using a UV Stratalinker 1800 (Stratagene). After prehybridization with ULTRAhyb buffer at 42°C for 1 hr, 4 μL of a DIG-labeled DNA probe specific to the Cd1 riboswitch or the 5S rRNA loading control gene was added. After 16 hours, the membrane washed in low and high stringency buffers sequentially (NorthernMax-Gly Kit). To detect the DIG-labeled probes on the membrane, we used the buffers and followed the manufacturer’s instructions in the DIG High Prime DNA Labeling and Detection Starter Kit II (Roche) and the chemiluminescent substrate CDP-Star (Roche). Membranes were exposed to film, which was then imaged using a developer.

### Detection of TcdA by western blot

*C*. *difficile* was grown as patches on BHIS agar for 24 hours. The patches were thoroughly suspended in BHIS, and cell densities were normalized to an OD_600_ 1.0–1.5. The cells were again pelleted by centrifugation and then suspended in 1X SDS-PAGE sample buffer [113] and heated at 95°C for 10 minutes. The lysates were separated on an 8% SDS-polyacrylamide gel then transferred to a nitrocellulose membrane (Bio-Rad). TcdA was detected using a mouse α-TcdA primary antibody (Novus Biologicals) and goat anti-mouse IgG conjugated with IR800 (Thermo Fisher) as described previously [44]. Detection was done using a LI-COR Odyssey Imager (LI-COR Biosciences). Three independent experiments were done, each with three to four biological replicates of *flg* ON and OFF isolates.

### Cell viability assay

Vero cells were seeded in 96 well plates at 8,000 cells per well in DMEM (Life Technologies) supplemented with 10% Fetal Bovine Serum (Sigma) (DMEM-FBS) and 1X Penicillin/Streptomycin (Life Technologies) for 16 hours. *C*. *difficile* R20291 *flg* ON and OFF isolates, and a *sigD* mutant control, were grown on BHIS plates for 16–24 hours. Four colonies of each isolate/strain were grown in TY medium overnight, then diluted 1:50 into fresh TY and grown to stationary phase (OD_600_ ≈ 2.0). Cultures were normalized to the same OD_600_. Bacteria were removed by centrifugation, and supernatants were collected, filter sterilized (0.45 micron), and serially diluted in TY medium. For each well with Vero cells, the cell culture medium was replaced with fresh DMEM-FBS. Supernatants were added in the following dilutions with cell culture media: 1:4, 1:40, 1:80, 1:400, and 1:800. As a negative control, Vero cells were treated with TY medium at a 1:4 dilution in DMEM-FBS. After 24 hours at 37°C, the medium was carefully removed by aspiration, and 100 μL each of cell culture medium and Promega CellTiter Glo reagent were added to each well. After 10 minutes at room temperature on a shaker, Vero cell lysates were transferred to opaque-walled 96 well plates. Luminescence was measured using a Synergy H1 Hybrid plate reader with Gen5 software (BioTek). Data were combined from two independent experiments each with four biological replicates of each isolate/strain.

### Identification of the recombinase that catalyzes inversion of the flagellar switch

Relevant *E*. *coli* strains are listed in S1 Table. Bacteria were passaged onto LB plates with ampicillin 50 μg/mL and kanamycin 50 μg/mL and grown overnight for single colonies. Bacteria were inoculated into LB with the above-mentioned antibiotics to grow overnight at 30°C, diluted 1:50 into fresh medium in duplicates, and grown to an OD_600_ 0.4. Anhydrotetracycline (ATc) was added to a biological replicate for each flagellar switch/recombinase pair at a final concentration of 200 ng/mL, grown until OD_600_ 1.0, and plasmids were purified using the GeneJET Plasmid Miniprep Kit (Thermo Fisher). Purified plasmids were used as template in an orientation-specific PCR assay to identify the recombinase that catalyzes inversion from ON to OFF and OFF to ON.

### Microscopy

For transmission electron microscopy, *C*. *difficile flg* ON and OFF populations were isolated on BHIS agar plates as described above and briefly washed in Dulbecco’s PBS (Sigma) prior to suspension in PBS-4% paraformaldehyde for fixation for 1 hour in the anaerobic chamber. Cell suspensions were adsorbed onto Formvar/copper grids, washed in water, and stained for 30 seconds in two sequential drops of 1.5% aqueous uranyl acetate. Cells were observed on a LEO EM 910 Transmission Electron Microscope (Carl Zeiss Microscopy, LLC, Thornword, NY) and recorded with a Gatan Orius SC1000 digital camera with Digital Micrograph 3.11.0 software (Gatan, Inc., Pleaston, CA). To image bacterial colony morphology, we used a Zeiss Stereo Discovery V8 dissecting microscope with a glass stage for illumination of bacterial colonies with light from the top and bottom.

For fluorescence microscopy, *C*. *difficile* enriched *flg* ON and OFF isolates and the *sigD* mutant bearing *mCherryOpt* reporter fusions (S1 Table) were cultured overnight in BHIS medium, diluted 1:50 in fresh BHIS medium, and grown to an OD_600_ of ~0.5. For strains with the ATc-inducible *mCherryOpt* reporter (pDSW1728), 15 ng/mL of ATc was added to the culture at OD_600_ ~0.3 to induce transcription of the *mCherryOpt* gene. One milliliter of each culture was briefly pelleted in the anaerobic chamber, washed and suspended in phosphate buffered saline (PBS), and fixed according to published methods in PBS and paraformaldehyde [73]. After 3 hours in fixative in the dark at 4°C, bacterial pellets were washed three times in PBS and suspended in 0.5 mL PBS with 3 μg/mL 4′,6-diamidino-2-phenylindole (DAPI) to label DNA. After overnight incubation at 4°C in the dark, samples were pelleted and suspended in 1 mL of PBS. Bacteria were immobilized on agar pads as previously described [73] and covered with 1.5 thickness glass cover slips (ThermoFisher) for fluorescence microscopy. The Olympus BX61 Upright Wide Field Microscope with a 60X/1.42 Oil PlanApo N objective lens was used for imaging samples, and Volocity 6.3 software was used for image acquisition. Multiple fields were taken for each sample in a coordinated fashion to ensure no repeated sections. Three images were automatically taken for each field in the differential contrast (DIC) channel, DAPI channel (Excitation: 377 nm (+/- 25 nm), Emission: 447 nm (+/- 30nm)), and Texas Red/RFP channel (Excitation: 562 nm (+/- 20 nm), Emission: 642 nm (+/- 20 nm)) with consistent settings for side-by-side comparison. Images were processed using the FIJI version of ImageJ [114], and bacteria were visually enumerated for +/- mCherryOpt fluorescence, with DAPI staining and DIC channels allowing imaging of all bacteria.

## Supporting information

S1 Table Strains and plasmids used in this study. (DOCX)

S2 Table Oligonucleotides used in this study. (DOCX)

S1 Methods Details of molecular cloning for strains and plasmids used in this study. (DOCX)

S1 Fig Spore stocks of *C*. *difficile* R20291 contain both *flg* ON and OFF bacteria. Boiled lysates of *C*. *difficile* R20291 spores served as the templates in an asymmetric PCR-digestion assay with primers R591 and R857 and the restriction enzyme SwaI. Shown are the results for two independent spore preparations.
(PDF)

S2 Fig Stability of the flagellar switch during growth in liquid and solid media. (A) Asymmetric PCR-digestion assay with template from enriched *C*. *difficile* R20291 *flg* ON and OFF isolates grown on an agar surface for 24 hours and 48 hours. T_0_ represents the cultures used to inoculate the agar plates. (B) Asymmetric PCR-digestion assay of *C*. *difficile* R20291 *flg* ON and OFF isolates grown in BHIS medium collected at T_0_, two exponential phase time points (EXP, OD_600_ 0.5 and 1.0), and two stationary phase time points (STAT, OD_600_ 1.8 and overnight, O/N). Images are representative from two independent experiments with four replicates of each *flg* phase. (C) Growth curve of *C*. *difficile* R20291 *flg* ON and OFF isolates in BHIS medium. Data are combined from two independent experiments each with two replicates of each *flg* phase, and means and standard deviations are shown.
(PDF)

S3 Fig Appearance of Smooth, Circular (SC) and Rough, Filamentous (RF) colony morphotypes. *C*. *difficile* R20291 spores were plated on BHIS agar supplemented with taurocholate to obtain single colonies. After 24 hours, individual colonies were suspended in media and spotted onto fresh BHIS plates. Every 24 hours for four days, colony spots were suspended, serially diluted, and plated on BHIS to enumerate colonies based on their morphologies. (A) We observed two distinct colony morphologies based on colony texture and edge—a smooth, circular (SC) morphotype, and a rough, filamentous (RF) morphotype. (B) The percentage of RF colonies increased, and the percentage of SC colonies decreased over time. Using the asymmetric PCR-digestion assay to determine the orientation of the flagellar switch in SC and RF colonies after 96 hours, colony morphology and switch orientation were determined to be unlinked. 72% of bacteria in the SC colonies had the flagellar switch in the published orientation, and 64% in the RF colonies had the switch in the inverse orientation. (C,D) Bacteria from SC and RF colony morphology maintained their respective characteristic morphologies after passaging.
(PDF)

S4 Fig Quantification and controls for fluorescence microscopy studies in Fig 4. (A) Frequency of RFP (mCherryOpt)-positive and -negative bacteria for strains evaluated in Fig 4D. Number of bacteria counted per group transformed with P_*flgM*_::*mCherryOpt*: *flg* ON (n = 3321 bacteria; 7 biological replicates), *flg* OFF (n = 3720 bacteria; 6 biological replicates), and *sigD* (n = 1348, 3 biological replicates). (B) Frequency of RFP (mCherryOpt)-positive and -negative bacteria of strains in panel C. Number of bacteria counted per group transformed with P_Tet_::*mCherryOpt*: *flg* ON (n = 662 bacteria; 3 biological replicates) and *flg* OFF (n = 401 bacteria; 3 biological replicates). Number of bacteria counted per group transformed with promoterless::*mCherryOpt*: *flg* ON (n = 964 bacteria; 3 biological replicates), *flg* OFF (n = 772 bacteria; 3 biological replicates), and *sigD* (n = 670, 3 biological replicates). (C) Representative fluorescence micrographs for the indicated strains containing fusions of *mCherryOpt* to the P*tet* promoter (grown with ATc, positive control) or promoterless *mCherryOpt* (negative controls). White bar = 10 microns. The channels used are indicated at the top.
(PDF)

S5 Fig Construction and confirmation of the *sigD*::*ermB* mutation in *C*. *difficile* R20291. (A). Schematic diagram of the Group II intron disruption of *sigD* in *C*. *difficile* R20291 (CDR20291_0270). The Targetron construct was designed previously to insert at nucleotide position 228 of the *sigD* gene in the sense orientation [60]. The *sigD* gene is 702 bp. The forward and reverse primers, R1887 (red arrow) and R1888 (green arrow), partially flank *sigD* and produce a PCR product of 716 bp. Insertion of the Group II intron into *sigD* (*sigD*::*ermB*) results in a PCR product of ~2500 bp. A second PCR reaction was used to confirm *sigD*::*ermB* by using R991, a Group II intron specific primer called EBS Universal. A PCR reaction with the R1887 and R991 yields a product of ~450 bp if the Group II intron is in *sigD*. (B). Image of an EtBr stained agarose gel with PCR products for the different reactions detailed in (A). Lane 1: Wildtype R20291 used as template with primers R1887 and R1888. Lane 2: The putative R20291 *sigD*::*ermB* mutant used as template with primers R1887 and R1888. Lane 3: The putative R20291 *sigD*::*ermB* mutant used as template with primers R1887 and R991.
(PDF)

S6 Fig Confirmation of flagellar switch orientation accompanying alkaline phosphatase assays in *C*. *difficile* and *B*. *subtilis*. (A, B) Asymmetric PCR-digestion assay to determine the orientation of the flagellar switch. For the native locus, we used primers R1705 and R1704 in PCR reactions. R1705 (forward primer) anneals 3’ of the Cd1 riboswitch DNA sequence, and R1704 (reverse primer) anneals to the second gene in the *flgB* operon, *flgC* (CDR20291_0249). For the reporter locus (B), we used R1705 and R1706, a reverse primer that anneals to the alkaline phosphatase gene, *phoZ*, for PCR amplification. Biological replicates for each AP reporter in *C*. *difficile* R20291 were combined and genomic DNA was extracted, based on published methods, to simultaneously determine the orientation of the native flagellar switch in each. The numerical designations for each reporter are shown in parentheses and correspond to Fig 7A. We confirmed that both the native and reporter flagellar switches for each AP reporter strain were in their expected orientations. (C) Orientation-specific PCR assay of AP reporters #3–6 in *B*. *subtilis* showing that the AP reporter flagellar switches maintained their orientations.
(PDF)

S7 Fig Alkaline phosphatase activity of the *phoZ* gene reporters in *C*. *difficile flg* OFF. Activity was assessed as described in the main text in tandem with the same reporters in the R20291 *flg* ON background.
(PDF)

S8 Fig Expression of *recV* in *C*. *difficile flg* ON and *flg* OFF isolates stimulates inversion of the flagellar switch. (A) Asymmetric PCR-digestion assay of products from *C*. *difficile* R20291 *flg* ON and OFF isolates transformed with pRT1611 (V, vector), or pRT1529 (pRPF185::*recV*), grown with or without 10 ng/ml ATc (S1 Table). (B) Increased transcription of *recV* occurs in *C*. *difficile* with pRT1529 in the absence of ATc induction. qRT-PCR measuring transcript abundance of *recV* and *codY* control gene in R20291 *flg* ON and OFF, each with pRT1611 (vector) and RT1529 (*recV*). Data were normalized to the *rpoC* gene and expressed relative to the respective parental strain with pRT1611. Shown are means and standard deviations.
(PDF)

S9 Fig Identification of *C*. *difficile* R20291 *recV flg* ON* mutants. *C*. *difficile* R20291 *recV flg* OFF* strain was transformed with a plasmid for expression of *recV* to allow flagellar switch inversion. The strain was then passaged without antibiotics to allow plasmid loss. Five thiamphenicol-sensitive colonies were identified and screened by PCR as in Fig 1 for the orientation of the *cwpV* and flagellar switches. Shown: orientation-specific PCR assay of five isolates, two of which (#1, #3) have the flagellar switch in the ON orientation; both have the *cwpV* switch in the OFF orientation.
(PDF)

S10 Fig Isolation of motile *recV flg* OFF* suppressor mutants. (A) Representative image of a motility assay after 72 hours, with *flg* ON/pP_*tet*_ (RT1615), *flg* OFF/pP_*tet*_ (RT1617), *sigD*/pP_*tet*_ (RT1690), *recV flg* ON*/pP_*tet*_ (RT1715), *recV flg* ON*/pP_*tet*_::*recV* (RT1716), *recV flg* OFF*/pP_*tet*_ (RT1691), and *recV flg* OFF*/pP_*tet*_::*recV* (RT1697). Strain numbers are listed in parentheses. The *recV flg* OFF*/pP_*tet*_::*recV* (RT1691) showed motility upon this prolonged incubation. (B) Orientation-specific PCR assay of the flagellar switch from *recV flg* ON*, *recV flg* OFF*, and three motile suppressor (MS) mutants of *recV flg* OFF* (pRT1719 –RT1724). Image representative of two independent experiments with eight biological replicates of motile suppressor mutants of *recV flg* OFF*.
(PDF)

S11 Fig Sequence alignment of the flagellar switch and inverted repeat sequences from NCBI accessible genomes of *C*. *difficile*. The following *C*. *difficile* genomes were used in a sequence alignment for the flagellar switch: 630, BI9, M68, CF5, R20291, CD196, BI1, and 2007855.
(PDF)
