# Supplementary material for: A genetic switch controls the production of flagella and toxins in Clostridium difficile
Source: PLoS Genet. 2017 Mar 27;13(3):e1006701. doi: 10.1371/journal.pgen.1006701 (PMC5386303; doi:10.1371/journal.pgen.1006701)
Supplement: S1 Table — (DOCX) [file pgen.1006701.s001.docx]

S1 Table. Strains and plasmids used in this study.

| Strain/Plasmid | Description/Purpose | Reference |
| --- | --- | --- |
| Plasmids |  |  |
| pMC123 | *E. coli* – *C. difficile* shuttle vector; Amp^R^, Cm^R^/Tm^R^ | [1] |
| pMC358 | pMC123 with *Enterococcus faecalis phoZ,* including native *phoZ* promoter | [2] |
| pRT1323 | pMC123::P*_flgB_*-5’UTR(FS^ON^)-*flgB::phoZ* pMC123 with *flgB* operon promoter, 5’ UTR with the flagellar switch in the ON orientation, and *flgB,* fused to *phoZ* | This work |
| pRT1324 | pMC123::P*_flgB_*-5’UTR(FS^OFF^)-*flgB::phoZ* pMC123 with *flgB* operon promoter, 5’ UTR with the flagellar switch in the OFF orientation, and *flgB,* fused to *phoZ* | This work |
| pSMB47 | Tn*916* integrational vector Erm^R^/CmR | [3] |
| pRT1532 | pSMB47::*slpATT;*  pSMB47 with the *C. difficile* 630 *slpA* Rho-independent terminator | This work |
| pRT1346 | Promoterless *phoZ* in pRT1532 (::*phoZ*) | This work |
| pRT1345 | *C. difficile* R20291 *flgB* operon promoter fused to *phoZ* in pRT1532 (P*_flgB_*::*phoZ*) | This work |
| pRT1326 | *C. difficile* R20291 *flgB* operon promoter, 5’ UTR with the flagellar switch in the ON orientation, and *flgB,* fused to *phoZ* in pRT1532 (P*_flgB_*-5’UTR(FS^ON^)-*flgB::phoZ*) | This work |
| pRT1344 | *C. difficile* R20291 *flgB* operon promoter, 5’ UTR with the flagellar switch in the OFF orientation, and *flgB,* fused to *phoZ* in pRT1532 (P*_flgB_*-5’UTR(FS^OFF^)-*flgB::phoZ*) | This work |
| pRT1254 | *C. difficile* R20291 flagellar switch in the ON orientation and *flgB*, fused to *phoZ* in pRT1532 (FS^ON^-*flgB::phoZ*) | This work |
| pRT1286 | *C. difficile* R20291 flagellar switch in the OFF orientation and *flgB* gene, fused to *phoZ* in pRT1532 (FS^OFF^-*flgB::phoZ*) | This work |
| pMWO-074 | Low copy anhydrotetracycline inducible expression vector, Kan^R^ | [4] |
| pRT1164 | pMWO-074::*recV* (CDR20291_1004) | This work |
| pRT1165 | pMWO-074::*xerD1* (CDR20291_1060) | This work |
| pRT1166 | pMWO-074::CDR20291_1068 | This work |
| pRT1167 | pMWO-074::*xerD2* (CDR20291_1174) | This work |
| pRT1221 | pMWO-074::CDR20291_1826 | This work |
| pRT1222 | pMWO-074::CDR20291_1855 | This work |
| pRT1223 | pMWO-074::CDR20291_1973 | This work |
| pRT1224 | pMWO-074::CDR20291_3416 | This work |
| pRPF185 | Anhydrotetracycline (ATc) inducible expression vector; Cm^R^/Tm^10^ | [5] |
| pRT1611 | β-glucuronidase gene*, gusA*, removed to generate reporterless derivative of pRPF185 | This work |
| pRT1529 | pRPF185::*recV* (CDR20291_1004) | This work |
| pBL100 | Targetron vector containing untargeted group II intron; Amp^R^ | [6] |
| pRT1073 | pBL100::*sigD*, for Targetron integration at nucleotide position 228 of *sigD* (CDR20291_0270), sense orientation | [7,8] |
| pDSW1728 | *mCherryOpt*; gene in MCS downstream of anhydrotetracycline-inducible promoter system derivative of pRPF185 (RT1685) | [9] |
| pRPF144 | *E. coli*-*C. difficile* shuttle vector with P*_cwp2_::gusA* cassette; Cm^R^/Tm^R^ | [5] |
| pRT1676 | pRPF144::P*_flgM_::mCherryOpt* (replaces P*_cwp2_::gusA* in pRPF144 with P*_flgM_*) | This work |
| pRT1483 | pRPF144::*mCherryOpt* (promoterless) | This work |
| pRT1719 | *E. coli* DH5α pCR4-TOPO: P*_flgB_-*5'UTR(FS^ON^)-*flgB* cloned from *C. difficile* R20291 RT1702 | This work |
| pRT1720 | *E. coli* DH5α pCR4-TOPO: P*_flgB_-*5'UTR(FS^OFF^)-*flgB* cloned from *C. difficile* R20291 RT1693 | This work |
| pRT1721 | *E. coli* DH5α pCR4-TOPO: P*_flgB_-*5'UTR(FS^OFF^)-*flgB* cloned from *C. difficile* R20291 RT1704 | This work |
| pRT1722 | *E. coli* DH5α pCR4-TOPO: P*_flgB_-*5'UTR(FS^OFF^)-*flgB* cloned from *C. difficile* R20291 RT1705 | This work |
| pRT1723 | *E. coli* DH5α pCR4-TOPO: P*_flgB_-*5'UTR(FS^OFF^)-*flgB* cloned from *C. difficile* R20291 RT1706 | This work |
| pRT1724 | *E. coli* DH5α pCR4-TOPO: P*_flgB_-*5'UTR(FS^OFF^)-*flgB* cloned from *C. difficile* R20291 RT1707 | This work |
|  |  |  |
| *Escherichia coli* Strains |  |  |
| DH5α | F- Φ80*lacZ* Δ M15 (*lacZYA-argF)*U169 *recA1* *endA1 hsdR*17(rκ -, mκ +) *phoA supE44 thi-*1 *gyrA*96 *relA1 λ ^-^ tonA* | Invitrogen, [10] |
| HB101(pRK24) | F^-^ *mcrB mrr hsdS20(r_B_^-^m_B_)recA13 leuB6 ara-14 proA2 lacY1 galK2 xyl-5 mtl-1 rpsL20* (pRK24) | [1] |
| RT1246 | *E. coli* DH5α co-transformed with pRT1323 and pRT1164 | This work |
| RT1247 | *E. coli* DH5α co-transformed with pRT1323 and pRT1165 | This work |
| RT1248 | *E. coli* DH5α co-transformed with pRT1323 and pRT1166 | This work |
| RT1249 | *E. coli* DH5α co-transformed with pRT1323 and pRT1167 | This work |
| RT1250 | *E. coli* DH5α co-transformed with pRT1323 and pRT1221 | This work |
| RT1251 | *E. coli* DH5α co-transformed with pRT1323 and pRT1222 | This work |
| RT1252 | *E. coli* DH5α co-transformed with pRT1323 and pRT1223 | This work |
| RT1253 | *E. coli* DH5α co-transformed with pRT1323 and pRT1224 | This work |
| RT1310 | *E. coli* DH5α co-transformed with pRT1324 and pRT1164 | This work |
| RT1311 | *E. coli* DH5α co-transformed with pRT1324 and pRT1165 | This work |
| RT1312 | *E. coli* DH5α co-transformed with pRT1324 and pRT1166 | This work |
| RT1313 | *E. coli* DH5α co-transformed with pRT1324 and pRT1167 | This work |
| RT1314 | *E. coli* DH5α co-transformed with pRT1324 and pRT1221 | This work |
| RT1315 | *E. coli* DH5α co-transformed with pRT1324 and pRT1222 | This work |
| RT1316 | *E. coli* DH5α co-transformed with pRT1324 and pRT1223 | This work |
| RT1317 | *E. coli* DH5α co-transformed with pRT1324 and pRT1224 | This work |
|  |  |  |
| *Bacillus subtilis* Strains |  |  |
| BS49 | CU2189::Tn916 for conjugation into *C. difficile*; Tet^R^ | [11] [12] |
| RT1392 | BS49 transformed with pRT1346 (promoterless *phoZ*) | This work |
| RT1393 | BS49 transformed with pRT1345 (P*_flgB_::phoZ*) | This work |
| RT1394 | BS49 transformed with pRT1326 (P*_flgB_*-5’UTR(FS^ON^)-*flgB::phoZ*) | This work |
| RT1395 | BS49 transformed with pRT1344 (P*_flgB_*-5’UTR(FS^OFF^)-*flgB::phoZ*) | This work |
| RT1331 | BS49 transformed with pRT1254 (FS^ON^-*flgB::phoZ*) | This work |
| RT1332 | BS49 transformed with pRT1286 (FS^OFF^-*flgB::phoZ*) | This work |
|  |  |  |
| *Clostridium difficile* Strains |  |  |
| 630*Δerm* | Ribotype 012, erythromycin susceptible derivative of *C. difficile* 630 | [13] |
| ATCC 43598 | Ribotype 017, gift from Shonna McBride | ATCC |
| R20291 | Ribotype 027, epidemic isolate | [14] |
| RT1566 | *C. difficile* R20291 with a Targetron insertion at nucleotide position 228 of *sigD*, sense orientation (*sigD::erm*) | This work |
| RT1536 | *C. difficile* R20291 *flg* ON isolate with promoterless *phoZ* fusion integrated on the chromosome via Tn916 (RT1392) | This work |
| RT1537 | *C. difficile* R20291 *flg* OFF isolate with promoterless *phoZ* fusion integrated on the chromosome via Tn916 (RT1392) | This work |
| RT1538 | *C. difficile* R20291 *flg* ON isolate with P*_flgB_::phoZ* fusion integrated on the chromosome via Tn916 (RT1393) | This work |
| RT1539 | *C. difficile* R20291 *flg* OFF isolate with P*_flgB_::phoZ* fusion integrated on the chromosome via Tn916 (RT1393) | This work |
| RT1540 | *C. difficile* R20291 *flg* ON isolate with P*_flgB_-*5’UTR(FS^ON^)-*flgB::phoZ* fusion integrated on the chromosome via Tn916 (RT1394) | This work |
| RT1541 | *C. difficile* R20291 *flg* OFF isolate with P*_flgB_*_-_5’UTR(FS^ON^)-*flgB::phoZ* fusion integrated on the chromosome via Tn916 (RT1394) | This work |
| RT1542 | *C. difficile* R20291 *flg* ON isolate with P*_flgB_-*5’UTR(FS^OFF^)-*flgB::phoZ* fusion integrated on the chromosome via Tn916 (RT1395) | This work |
| RT1543 | *C. difficile* R20291 *flg* OFF isolate with P*_flgB_-*5’UTR(FS^OFF^)-*flgB::phoZ* fusion integrated on the chromosome via Tn916 (RT1395) | This work |
| RT1544 | *C. difficile* R20291 *flg* ON isolate with FS^ON^-*flgB::phoZ* fusion integrated on the chromosome via Tn916 (RT1331) | This work |
| RT1545 | *C. difficile* R20291 *flg* OFF isolate with FS^ON^-*flgB::phoZ* fusion integrated on the chromosome via Tn916 (RT1331) | This work |
| RT1546 | *C. difficile* R20291 *flg* ON isolate with FS^OFF^-*flgB::phoZ* fusion integrated on the chromosome via Tn916 (RT1332) | This work |
| RT1547 | *C. difficile* R20291 *flg* OFF isolate with FS^OFF^-*flgB::phoZ* fusion integrated on the chromosome via Tn916 (RT1332) | This work |
| RT1615 | *C. difficile* R20291 *flg* ON isolate with pRT1611 | This work |
| RT1616 | *C. difficile* R20291 *flg* ON isolate with pRT1529 | This work |
| RT1617 | *C. difficile* R20291 *flg* OFF isolate with pRT1611 | This work |
| RT1618 | *C. difficile* R20291 *flg* OFF isolate with pRT1529 | This work |
| RT1690 | *C. difficile* R20291 *sigD::ermB* with pRT1611 | This work |
| RT1689 | *C. difficile* R20291 *sigD::ermB* with pRT1676 | This work |
| RT1695 | *C. difficile* R20291 *flg* ON isolate with pRT1676 | This work |
| RT1696 | *C. difficile* R20291 *flg* OFF isolate with pRT1676 | This work |
| RT1698 | *C. difficile* R20291 *flg* ON isolate with pDSW1728 | This work |
| RT1699 | *C. difficile* R20291 *flg* OFF isolate with pDSW1728 | This work |
| RT1712 | *C. difficile* R20291 *flg* ON isolate with pRT1483 | This work |
| RT1713 | *C. difficile* R20291 *flg* OFF isolate with pRT1483 | This work |
| RT1714 | *C. difficile* R20291 *sigD::ermB* with pRT1483 | This work |
| RT1693 | *C. difficile* R20291 *recV*::*ermB* (*cwpV* OFF; *flg* OFF)^a^ | [15] |
| RT1694 | *C. difficile* R20291 *recV*::*ermB* (*cwpV* ON; *flg* OFF)^a^ | [15] |
| RT1691 | *C. difficile* R20291 *recV::ermB* (*cwpV* OFF; *flg* OFF)^a^ with pRT1611 | This work |
| RT1697 | *C. difficile* R20291 *recV*::*ermB* (*cwpV* OFF; *flg* OFF)^a^ with pRT1529 | This work |
| RT1702 | *C. difficile* R20291 *recV::ermB* (*cwpV* OFF; *flg* ON)^a^ | This work |
| RT1715 | *C. difficile* R20291 *recV::ermB* (*cwpV* OFF; *flg* ON)^a^ with pRT1611 | This work |
| RT1716 | *C. difficile* R20291 *recV::ermB* (*cwpV* OFF; *flg* ON)^a^ with pRT1529 | This work |
| RT1704 | *C. difficile* R20291 *recV::ermB* *flg* OFF motile suppressor mutant #1 | This work |
| RT1705 | *C. difficile* R20291 *recV::ermB* *flg* OFF motile suppressor mutant #2 | This work |
| RT1706 | *C. difficile* R20291 *recV::ermB* *flg* OFF motile suppressor mutant #3 | This work |
| RT1707 | *C. difficile* R20291 *recV::ermB* *flg* OFF motile suppressor mutant #4 | This work |

^a­^Genotype in parentheses reflects starting orientations of the indicated switches.

**References:**

1. McBride SM, Sonenshein AL. Identification of a genetic locus responsible for antimicrobial peptide resistance in *Clostridium difficile*. Infect Immun. 2011 Jan;79(1):167–76.

2. Edwards AN, Pascual RA, Childress KO, Nawrocki KL, Woods EC, McBride SM. An alkaline phosphatase reporter for use in *Clostridium difficile*. Anaerobe. 2015 Jan 7;32C:98–104.

3. Manganelli R, Provvedi R, Berneri C, Oggioni MR, Pozzi G. Insertion vectors for construction of recombinant conjugative transposons in *Bacillus subtilis* and *Enterococcus faecalis*. FEMS Microbiol Lett. 1998 Nov 15;168(2):259–68.

4. Obrist MW, Miller VL. Low copy expression vectors for use in Yersinia sp. and related organisms. Plasmid. 2012 Jul;68(1):33–42.

5. Fagan RP, Fairweather NF. *Clostridium difficile* Has Two Parallel and Essential Sec Secretion Systems. J Biol Chem. 2011 Jan 5;286(31):27483–93.

6. Bouillaut L, Self WT, Sonenshein AL. Proline-dependent regulation of *Clostridium difficile* Stickland metabolism. J Bacteriol. 2013 Feb;195(4):844–54.

7. Bordeleau E, Purcell EB, Lafontaine DA, Fortier L-C, Tamayo R, Burrus V. Cyclic di-GMP riboswitch-regulated type IV pili contribute to aggregation of *Clostridium difficile*. J Bacteriol. 2015 Mar;197(5):819–32.

8. Meouche El I, Peltier J, Monot M, Soutourina O, Pestel-Caron M, Dupuy B, et al. Characterization of the SigD regulon of *C. difficile* and its positive control of toxin production through the regulation of *tcdR*. PLoS ONE. 2013;8(12):e83748.

9. Ransom EM, Ellermeier CD, Weiss DS. Use of mCherry Red Fluorescent Protein for Studies of Protein Localization and Gene Expression in *Clostridium difficile*. Appl Environ Microbiol. 2015 Mar 1;81(5):1652–60.

10. Hanahan D. Studies on transformation of *Escherichia coli* with plasmids. Journal of Molecular Biology. 1983 Jun;166(4):557–80.

11. Christie PJ, Korman RZ, Zahler SA, Adsit JC, Dunny GM. Two conjugation systems associated with *Streptococcus faecalis* plasmid pCF10: identification of a conjugative transposon that transfers between *S. faecalis* and *Bacillus subtilis*. J Bacteriol. 1987 Jun;169(6):2529–36.

12. Browne HP, Anvar SY, Frank J, Lawley TD, Roberts AP, Smits WK. Complete genome sequence of BS49 and draft genome sequence of BS34A, *Bacillus subtilis* strains carrying Tn916. FEMS Microbiol Lett. 2015 Jan;362(3):1–4.

13. Hussain HA, Roberts AP, Mullany P. Generation of an erythromycin-sensitive derivative of *Clostridium difficile* strain 630 (630Δerm) and demonstration that the conjugative transposon Tn916ΔE enters the genome of this strain at multiple sites. J Med Microbiol. 2005 Feb 1;54(2):137–41.

14. Stabler RA, He M, Dawson L, Martin M, Valiente E, Corton C, et al. Comparative genome and phenotypic analysis of *Clostridium difficile* 027 strains provides insight into the evolution of a hypervirulent bacterium. Genome Biol. 2009;10(9):R102.

15. Sekulovic O, Ospina Bedoya M, Fivian-Hughes AS, Fairweather NF, Fortier L-C. The *Clostridium difficile* cell wall protein CwpV confers phase-variable phage resistance. Molecular Microbiology. 2015 Oct;98(2):329–42.
