# Supplementary material for: A genetic switch controls the production of flagella and toxins in Clostridium difficile
Source: PLoS Genet. 2017 Mar 27;13(3):e1006701. doi: 10.1371/journal.pgen.1006701 (PMC5386303; doi:10.1371/journal.pgen.1006701)
Supplement: S2 Table — (DOCX) [file pgen.1006701.s002.docx]

Table S2. Primers used in this study.

| Primer Name^a^ | Sequence (5’ 🡪 3’)^b^ | Lab Annotation |
| --- | --- | --- |
| *flg*Ribo012_Pub | GAGCAACTTTTCGAAGAAATATTTAAATAC | R1751 |
| *flg*Ribo012_Inv | GTATTTAAATATTTCTTCGAAAAGTTGCTC | R1752 |
| *flg*Ribo017_Pub | GAGCAACTTTTTGAAGAAATATTTAAATAC | R1622 |
| *flg*Ribo017_Inv | GTATTTAAATATTTCTTCAAAAAGTTGCTC | R1623 |
| *flg*Ribo027_Pub | AGGCAACTTTATAAAGAAATATTTAAATTTATATTAAAATATTTTTATATTTTTATTAGG | R1614 |
| *flg*Ribo027_Inv | CCTAATAAAAATATAAAAATATTTTAATATAAATTTAAATATTTCTTTATAAAGTTGCCT | R1615 |
| *flgB*Conserv_Rev | AGGCATAGCATCATTTAGTGTTTCTTC | R857 |
| *flg*UTR5’end_For | C**GAATTC**GTATACTTAAGTTAAAACTAAATAGGCAAATC (**EcoRI**) | R591 |
| EF2973_phoZRBSF | GTT**GAATTC**AGGAGGAAACAAGGAAATGAAG (**EcoRI**) | R1609 |
| EF2973_phoZCDSR | CAAGTT**GGATCC**CGTTCTGCTTTTTCTTCATTTTG (**BamHI**) | R1610 |
| CDR202_PflgBnew | GTTCAA**GCATGC**GATATATTGTACAAATAAAATTGAAATATATGG (**SphI**) | R1512 |
| CDR20291_0248CDSR | CAA**GAATTC**TTACCTCCCACTTATTATTGA | R1611 |
| CDR20291_asyPCRF | CACCTATAATAACATATTATACCAAATATAATTTAAAATAAAG | R1705 |
| *slpA*Term_F | GCGCACC**GGATCC**TATAAGT (**BamHI**) | R1848 |
| *slpA*Term_R | TGACTC**AAGCTT**CATCTTTTTATTTAGG (**HindIII**) | R1849 |
| EF2973_phoZRBSF | GTTCAA**GCATGC**AGGAGGAAACAAGGAAATGAAG (**SphI**) | R1632 |
| CDR20291_0248PR | GTT**GAATTC**AACTTAAGTATACAATAAATAAC (**EcoRI**) | R1608 |
| CDR20291_5'flgLIR | GTCAA**GCATGC**CACCTATAATAACATATTATACC (**SphI**) | R1673 |
| phoZ_AsymR | GAATGTTAATAAGGTAACCCCTAGCAAAGCTCTTTTCTTC | R1706 |
| CDR20291_0249R | GCTGTTAATCCACTAGCAGATATTCTC | R1704 |
| CDR20291_1004F (*recV*) | GTCAA**GGTACC**GCAACAAGACCTATAGAAATAG (**KpnI**) | R1675 |
| CDR20291_1004R (*recV*) | CAGTT**AAGCTT**TTAACCAATAAAGAAATTTTCAC (**HindIII**) | R1676 |
| CDR20291_1060F | GTCAA**GGTACC**GATATTATAGAGGGATATATAGATTAC (**KpnI**) | R1677 |
| CDR20291_1060R | CAGTT**AAGCTT**CTATTTAGCTTCTGCTTTTTGTAATTCTAC (**HindIII**) | R1678 |
| CDR20291_1068F | GTCAA**GGTACC**AAAGACCAAGGTATCATATTAGAAAC (**KpnI**) | R1679 |
| CDR20291_1068R | CAGTT**AAGCTT**TTACTGGTCTTCATGTAATTTCAAATGAAC (**HindIII**) | R1680 |
| CDR20291_1174F | GTCAA**GGTACC**AAAAGGAAAGGAGTTATTATG (**KpnI**) | R1681 |
| CDR20291_1174R | CAGTT**AAGCTT**CTATTTTTTATTTATATCCAAATTATC (**HindIII**) | R1682 |
| CDR20291_1826F | GTCAA**GGTACC**AAAGCTGCAATTTATTCAAG (**KpnI**) | R1683 |
| CDR20291_1826R | CAGTT**GGATCC**CTAATTGCTTAAAGAAACAGAATTATCCA (**BamHI**) | R1684 |
| CDR20291_1855F | GTCAA**GGTACC**AATAATGATAAGAAAATTATAAAAGTAC (**KpnI**) | R1685 |
| CDR20291_1855R | CAGTT**AAGCTT**TTAATCGTCCTCAATCCATTCAAAATTC (**HindIII**) | R1686 |
| CDR20291_1973F | GTCAA**GGTACC**TATAAAATTTTGATTGGTGGGTTATATATG (**KpnI**) | R1687 |
| CDR20291_1973R | CAGTT**GGATCC**TTAATTAGCATTAAATAAACTTTCAG (**BamHI**) | R1688 |
| CDR20291_3416F | GTCAA**GGTACC**ATTATTATATATGGATATTGC (**KpnI**) | R1689 |
| CDR20291_3416R | CAGTT**AAGCTT**CTAAATTGATTTTTTAGCACGAATAAGTG (**HindIII**) | R1690 |
| CDR20291_1115qF (*codY*) | ATTAGGAACATTGGTACTTTCAAGAT | R910 |
| CDR20291_1115qR (*codY*) | TTGAACTACAGCTTTCTTTCTCATTT | R911 |
| CDR20291_0248qF (*flgB*) | GCAACTAATCTAAGAAGTCAGACAATAGC | R856 |
| CDR20291_0248qR (*flgB*) | AGGCATAGCATCATTTAGTGTTTCTTC | R857 |
| CDR20291_0270qF (*sigD*) | GAATATGCCTCTTGTAAAGAGTATAGCA | R860 |
| CDR20291_0270qR (*sigD*) | TGCATCAATCAATCCAATGACTCC | R861 |
| CDR20291_0227qF (autolysin) | CATCTGGATTTGATATGATTATG | R1671 |
| CDR20291_0227qR (autolysin) | AATCTACTAGCTGTATTATTTACT | R1672 |
| CDR20291_0230qF (*flgM*) | AAGGAAATGGCAAGTGTG | R1669 |
| CDR20291_0230qR (*flgM*) | TTATCCTCGCATATCCTCT | R1670 |
| CDR20291_0240qF (*fliC*) | CAAAGTAAGTCTATGGAGAA | R1584 |
| CDR20291_0240qR (*fliC*) | ACAGATATACCATCTTGAAC | R1585 |
| CDR20291_0581qF (*tcdR*) | AGCAAGAAATAACTCAGTAGATGATT | R908 |
| CDR20291_0581qR (*tcdR*) | TTATTAAATCTGTTTCTCCCTCTTCA | R909 |
| CDR20291_0584qF (*tcdA*) | GGAGAAGTCAGTGATATTGCTCTTG | R852 |
| CDR20291_0584qR (*tcdA*) | CAGTGGTAGAAGATTCAACTATAGCC | R853 |
| CDR20291_0582qF (*tcdB*) | AAGGAATATCTAGTTACAGAAGTATTAGAGC | R854 |
| CDR20291_0582qR (*tcdB*) | GCAGTGTCATTTATTTGACCTCCA | R855 |
| CDR20291_1004qF (*recV*) | TTACATACTGGCTTTAC | R1799 |
| CDR20291_1004qR (*recV*) | GACCTAAATTAGCTTCT | R1800 |
| CDR20291_r03NorthF | GCAAAGAGGATACACCTGT | R1795 |
| CDR20291_r03NorthR | GGCTACGTCCTACTCTCCCA | R1796 |
| Cd1Ribo_NorthF | GCAAATCTAGAGAAATCTAGTGACG | R1791 |
| Cd1Ribo_NorthR | CGTATACAATATACCTAGAACTCTT | R1792 |
| recV_RT709F | GAGTCA**GAGCTC**CAAATCAAACTAAGAGGAGTGGTTGAAA (**SacI**) | R1853 |
| recV_RT709R | TGACTC**GGATCC**ACGTTATAATTAACCAATAAAG (**BamHI**) | R1854 |
| CDR20291_0270F | GTAGTTAATGAATAGAGAAGAATTAAT | R1887 |
| CDR20291_0270R | TCACCATCTATATAGAATATTTAAG | R1888 |
| EBSuniv | CGAAATTAGAAACTTGCGTTCAGTAAAC | R991 |
| PflgM_F_NheI | CAAT**GCTAGC**TATGCGTGAATTATAT (**NheI**) | R2117 |
| R202flgMpromo_R | CTAAC**GAGCTC**TAATTTAATTATCGCTC (**SacI**) | R2046 |
| CwpVIS_R202F | CAAAACCATGTTTTTTATAACAATTCATTAAC | R1920 |
| CwpVIS_R202R | GTTAATGAATTGTTATAAAAAACATGGTTTTG | R1921 |
| CwpVqR | AGCATCTGCTATAGATGAGTCGTTT | R1050 |
| qPCR_FlgSwit-ON | GTTTTCTTACCAAAGTGATACATTATTATATTAATG | R2175 |
| qPCR_FlgSwit-OFF | CATTAATATAATAATGTATCACTTTGGTAAGAAAAC | R2176 |
| qPCR_FlgSwit-REV | GCTATTGTCTGACTTCTTAAATTAGTTGCAT | R2177 |
| pCR4-TOPO T3_F | ATTAACCCTCACTAAAGGGA |  |
| pCR4-TOPO T3_R | TAATACGACTCACTATAGGG |  |

^a^ Where locus tags are used in the primer name, the corresponding gene name is noted in parentheses

^b^ Restriction site sequences are highlighted with bold text; the corresponding enzyme is listed in parentheses.
