# Supplementary material for: A genetic switch controls the production of flagella and toxins in Clostridium difficile
Source: PLoS Genet. 2017 Mar 27;13(3):e1006701. doi: 10.1371/journal.pgen.1006701 (PMC5386303; doi:10.1371/journal.pgen.1006701)
Supplement: S1 Methods — (DOCX) [file pgen.1006701.s003.docx]

**A Genetic Switch Controls the Production of Flagella and Toxins in *Clostridium difficile***

Brandon R. Anjuwon-Foster and Rita Tamayo

**SUPPLEMENTAL METHODS**

**Generation of standard curve using known quantities of *flg* ON and OFF DNA**

The region spanning the promoter of the *flgB* operon, the 5’ UTR, and the *flgB* coding sequence was amplified by PCR from genomic DNA of *C. difficile* R20291 *flg* ON and OFF isolates using primers R1512 and R1611. The resulting 1063 bp products, P*_flgB_*-5’UTR(FS^ON/OFF^)-*flgB*, were digested with BamHI and EcoRI. The *phoZ* gene was PCR amplified from pMC358 template using primers R1609 and R1610. The 1473 bp product was digested with EcoRI and SphI. The digested P*_flgB_*-5’UTR(FS^ON/OFF^)-*flgB* and *phoZ* fragments were ligated into BamHI- and SphI-digested pMC123 to generate *flg* ON (pRT1323) and OFF (pRT1324) reporter plasmids for the standard curve. Preparations of these plasmids were mixed in known ratios of OFF/ON plasmids (0:100, 10:90, 20:80, 30:70, 40:60, 50:50, 60:40, 70:30, 80:20, 90:10, 100:0) for the asymmetric PCR-digestion assay. The mixtures were used as templates in PCR reactions with R591 and R857 to amplify the flagellar switch region. The PCR products were column purified, digested with the restriction enzyme SwaI, and electrophoresed in a 2.5% agarose gel. Images of the EtBr-stained gels were collected under UV using the G:BOX Chemi Imaging system, and band intensities in each lane were determined using Image J, as previously described [1].

**Construction of alkaline phosphatase reporters for use in *B. subtilis* and *C. difficile***

The *slpA* transcriptional terminator sequence was amplified from pRPF185 with R1848 and R1849, resulting in a 161 bp product that was digested with BamHI and HindIII and ligated into similarly digested pSMB47 to generate pRT1532. Alkaline phosphatase reporter fusions were subsequently cloned into this vector, 5’ of the transcriptional terminator. The full length reporters (P*_flgB_*-5’UTR(FS^ON/OFF^)-*flgB::phoZ*) were amplified from plasmids RT1323 and RT1324 with primers R1512 and R1610. The resulting 2530 bp product was digested with SphI and BamHI, and ligated into similarly digested pRT1532. The promoterless *phoZ* construct was made by PCR amplifying *phoZ* from pMC358 with primers R1632 and R1610, digesting the 1479 bp product with SphI and BamHI, and ligating into similarly digested pRT1532. The P*_flgB_::phoZ* reporter was made by PCR amplifying the *flgB* operon promoter region from *C. difficile* R20291 genomic DNA with primers R1512 and R1608 (250 bp product digested with SphI and EcoRI) and *phoZ* from pMC358 with primers R1609 and R1610 (1476 bp product digested with EcoRI and BamHI). Digested products for P*_flgB_* and *phoZ* were ligated into BamHI- and SphI-digested pRT1532. To generate fusions of the flagellar switch only (FS^ON/OFF^) to *phoZ*, the region downstream of the Cd1 riboswitch was amplified by PCR with primers R1673 and R1610 using pRT1323 and pRT1324 as templates for the *flg* ON and OFF orientations, respectively. The 2109 bp PCR products were digested with SphI and BamHI and ligated into similarly digested pRT1532. Purified plasmids for each reporter were transformed into *Bacillus subtilis* BS49, as previously described [1,2], and are listed in Table S1. Transformants with a flagellar switch were screened by orientation-specific PCR with R1614/1615 and R1706 to ensure that the expected orientation of ON or OFF was present and maintained. BS49 strains were conjugated with *C. difficile* R20291, and transformants were selected for lincomycin and tetracycline resistance, as previously described [2]. *C. difficile* transformants were screened for the Tn916 AP reporter by PCR and for the orientation of the native flagellar switch and the Tn916 AP reporter flagellar switch using the asymmetric PCR-digestion assay.

**Cloning of the putative recombinase genes for expression in *E. coli* and *C. difficile***

The genes for the eight recombinases conserved in the *C. difficile* genomes are listed with the R20291 locus tag: CDR20291_1004 (*recV*), CDR20291_1060, CDR20291_1068, CDR20291_1174, CDR20291_1826, CDR20291_1855, CDR20291_1973, and CDR20291_3416. The genes were amplified from *C. difficile* R20291 genomic DNA with primers named according to the pattern “locus tag F” and “locus tag R” (Table S2). PCR products were digested with KpnI and HindIII, except PCR products for CDR20291_1826 and CDR20291_1973 were digested with KpnI and BamHI. All PCR products were ligated into similarly digested pMWO-074 and transformed into *E. coli* DH5α. Sequence integrity was confirmed by sequencing the insertion in each resulting plasmid. Plasmids were purified from DH5α and co-transformed with either P*_flgB_*-5’UTR(FS^ON^)-*flgB::phoZ* (RT1323) or P*_flgB_*-5’UTR(FS^OFF^)-*flgB::phoZ* (RT1324) into *E. coli* DH5α to generate strains with a plasmid encoding one conserved recombinase and with a plasmid containing the flagellar switch in the ON or OFF orientation (Table S1).

To express *recV* in *C. difficile*, the *C. difficile* R20291 *recV* gene was PCR amplified from pRT1164 using primers R1853 and R1854. The 628 bp PCR product was digested with SacI and BamHI, ligated into similarly digested pRPF185, and transformed into *E. coli* DH5α, yielding strain pRT1529. The purified plasmid was transformed into *E. coli* HB101(pRK24) to allow transfer by conjugation of the *recV* expression plasmid into *C. difficile* R20291 *flg* ON and OFF isolates. Thiamphenicol and kanamycin resistant *C. difficile* transconjugants were selected for evaluation [2]. A control plasmid of the ATc-inducible expression vector was generated by removing the *gusA* gene from pRPF185 by digestion with SacI and BamHI and religating the vector backbone following treatment with Klenow. The vector was introduced into *C. difficile* R20291 *flg* ON and OFF isolates via conjugation with *E. coli* HB101(pRK24), and thiamphenicol and kanamycin resistance colonies were selected [2]. The presence of plasmids in *C. difficile* was confirmed by PCR with plasmid-specific primers flanking the multiple cloning site.

**Generation of the *C. difficile* R20291 *sigD::ermB* mutant**

To generate the *sigD::ermB* mutant in R20291, we used the previously generated retargeting plasmid pBL100::*sigD* (pRT1073), which inserts the Group II intron targeting sequence into *sigD* after nucleotide position 228 [3]. pRT1073 was transformed into *E. coli* HB101(pRK24) to allow its transfer into *C. difficile* R20291 via conjugation. Thiamphenicol- and kanamycin-resistant transconjugants were selected and passaged on BHIS-agar with lincomycin to identify clones with an insertion of the Group II intron. Candidate mutants were screened by PCR with primers for the *sigD* gene and for the Group II intron as described in S3 Figure.

**Construction of *mCherryOpt* reporter fusions and introduction into *C. difficile***

The pDSW1728 plasmid with the *mCherryOpt* gene under the transcriptional control of an anhydrotetracycline-inducible promoter (gifted by Dr. Craig Ellermeier) served as the base for reporter construction [4,5]. To generate pP*_flgM_::mCherryOpt*, P*_flgM_* was PCR amplified from R20291 genomic DNA using primers R2117 and R2046 and digested with NheI and SacI to generate a 51 bp product. The NheI and SacI digested P*_flgM_* was ligated into similarly digested pRPF144. The *mCherryOpt* gene was digested from pDSW1728 with SacI and BamHI. The 739 bp product was ligated into similarly digested pRPF144 with P*_flgM_* , yielding pRT1676. To generate the promoterless vector control plasmid (p*::mCherryOpt*), pP*_flgM_::mCherryOpt* was digested with NheI and SacI to remove the P*_flgM_*, and the vector backbone was treated with Klenow before being religated to yield pRT1685. The plasmids were confirmed by PCR and sequencing of the insertions, then transformed into *E. coli* HB101(pRK24) to allow conjugation with *C. difficile* R20291 enriched *flg* ON and *flg* OFF, and *sigD::ermB* as appropriate. Thiamphenicol- and kanamycin-resistant colonies were selected, and plasmids were detected by PCR with vector specific primers.

**Generation of *C. difficile recV* mutant derivatives**

The *C. difficile* R20291 *recV::ermB* *cwpV* ON* and OFF* (* locked) were obtained from Dr. Louis-Charles Fortier [6]. Orientation-specific PCR (Fig 1A) was performed to determine the orientation of the flagellar switch using primers R1614 and R857 for the ON orientation (375 bp) and R1615 and R857 for the OFF orientation (281 bp). Both *recV::ermB* isolates were found to be *flg* OFF* (Fig 8). To obtain a *recV::ermB* *flg* ON* isolate, we took advantage of the facts that pRPF185*::recV* (pRT1529) has leaky transcription in the absence of inducer, and that low levels of RecV are sufficient to promote a mixed population of *flg* ON and OFF bacteria (S8 Fig). The *C. difficile* R20291 *recV::ermB* *cwpV* OFF* and *flg* OFF* isolate (RT1693) was conjugated with *E. coli* HB101(pRK24) transformed with pRT1529 to obtain thiamphenicol and kanamycin resistant transconjugant RT1697. RT1697 was passaged in TY broth medium, diluting every 24 hours for 4 days in the absence of thiamphenicol selection to allow plasmid loss and to obtain a mixture of genetic switch orientations. Broth cultures were sampled on the 4^th^ day and cultured on plain BHIS agar to obtain single colonies. Eight single colonies were replica plated on BHIS +/- thiamphenicol plates. Colonies that were sensitive to thiamphenicol (indicating plasmid loss) were evaluated for the orientations of the flagellar and *cwpV* switches by orientation-specific PCR. The *cwpV* switch orientation was determined to identify a *cwpV* OFF* isolate, to prevent implication of *cwpV* in any phenotypes attributed to the flagellar switch. Primers R1920 and R1050 were used to detect the *cwpV* switch in the OFF orientation (493 bp product), while primers R1921 and R1050 were used to detect the *cwpV* ON orientation (346 bp product). Two *recV::ermB* *flg* ON*/*cwpV* OFF* clones were obtained, RT1702 and RT1703. Complementation of the *recV::ermB flg* ON* mutant was achieved by conjugation with *E. coli* HB101(pRK24) transformed with pRPF185::recV (pRT1529) and isolateion of thiamphenicol and kanamycin resistant transconjugants.

**Constriction of *C. difficile* strains for swimming motility assay and western blot**

*E. coli*(pRK24) transformed with pRT1611 (reporterless derivative of pRPF185) was conjugated with the following *C. difficile* R20291 *strains*: enriched *flg* ON, enriched *flg* OFF, *sigD::ermB*, *recV::ermB flg* ON* (*cwpV* OFF*), and *recV::ermB* *flg* OFF* (*cwpV* OFF*). Thiamphenicol and kanamycin resistant *C. difficile* transconjugants were selected for evaluation. These strains are listed in the Table S1 and were used in the swimming motility assay and western blot for TcdA in Figure 9 with *recV* mutants complemented with *recV* on as plasmid.

**Evaluation of the *flgB* operon regulatory region from *recV::ermB flg* OFF* motile suppressors**

The region upstream of the *flgB* operon consisting of the promoter and 5’UTR was amplified by PCR using primers R1512 and R1611 to generate a 1066 bp product from the following *C. difficile* R20291 *recV::ermB* strains: *flg* ON*, *flg* OFF*, and the eight *flg* OFF* motile suppressor mutants. The 1066 bp product was cloned into the pCR4-TOPO vector (ThermoFisher), as described by the manufacturer’s instructions, transformed into *E. coli* DH5α, and selected on agar medium for ampicillin resistance and loss of 5-bromo-4-chloro-3-indolyl-β-D-galactopyranoside (X-Gal) activity. White colonies were screened by colony lysis PCR for insertion of the 1066 bp into the vector using vector specific primers. Plasmid was purified from positive transformants and used as template in orientation-specific PCR to evaluate the orientation of the flagellar switch. PCR products were resolved in a 2.5% agarose gel and stained with EtBr for visualization. The region of interest was also sequenced using plasmid specific primers that flank the 1066 bp regulatory region and *flgB* gene. Sequencing results for the 1066 bp region from the eight *recV::ermB flg* OFF* suppressor mutants showed neither single nucleotide mutations nor inversion of the flagellar switch to the ON orientation compared to *recV::ermB flg* OFF*.

**References**

1. Lim JK, Gunther NW, Zhao H, Johnson DE, Keay SK, Mobley HL. In vivo phase variation of Escherichia coli type 1 fimbrial genes in women with urinary tract infection. Infect Immun. 1998 Jul;66(7):3303–10.

2. Bouillaut L, McBride SM, Sorg JA. Genetic Manipulation of Clostridium difficile. Hoboken, NJ, USA: John Wiley & Sons, Inc; 2005.

3. Bordeleau E, Purcell EB, Lafontaine DA, Fortier L-C, Tamayo R, Burrus V. Cyclic di-GMP riboswitch-regulated type IV pili contribute to aggregation of Clostridium difficile. J Bacteriol. 2015 Mar;197(5):819–32.

4. Ransom EM, Ellermeier CD, Weiss DS. Use of mCherry Red Fluorescent Protein for Studies of Protein Localization and Gene Expression in Clostridium difficile. Appl Environ Microbiol. 2015 Mar 1;81(5):1652–60.

5. Ransom EM, Weiss DS, Ellermeier CD. Use of mCherryOpt Fluorescent Protein in Clostridium difficile. Methods Mol Biol. 2016;1476:53–67.

6. Sekulovic O, Ospina Bedoya M, Fivian-Hughes AS, Fairweather NF, Fortier L-C. The Clostridium difficile cell wall protein CwpV confers phase-variable phage resistance. Molecular Microbiology. 2015 Oct;98(2):329–42.
