# Supplementary material for: A genetic switch controls the production of flagella and toxins in Clostridium difficile
Source: PLoS Genet. 2017 Mar 27;13(3):e1006701. doi: 10.1371/journal.pgen.1006701 (PMC5386303; doi:10.1371/journal.pgen.1006701)
Supplement: S1 Fig — Boiled lysates of C. difficile R20291 spores served as the templates in an asymmetric PCR-digestion assay with primers R591 and R857 and the restriction enzyme SwaI. Shown are the results for two independent spore preparations. (PDF) [file pgen.1006701.s004.pdf]

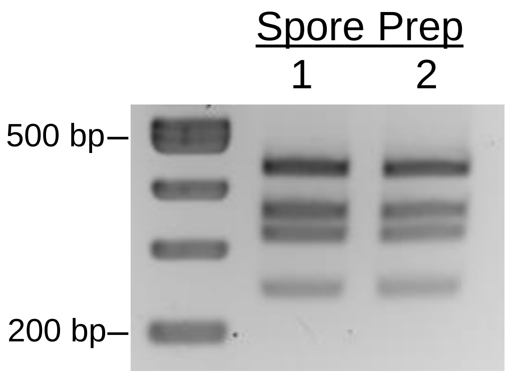

S1 Fig. Spore stocks of *C. difficile* R20291 contain both *flg* ON and OFF bacteria. Boiled lysates of *C. difficile* R20291 spores served as the templates in an asymmetric PCR-digestion assay with primers R591 and R857 and the restriction enzyme SwaI. Shown are the results for two independent spore preparations.
