# Supplementary material for: A genetic switch controls the production of flagella and toxins in Clostridium difficile
Source: PLoS Genet. 2017 Mar 27;13(3):e1006701. doi: 10.1371/journal.pgen.1006701 (PMC5386303; doi:10.1371/journal.pgen.1006701)
Supplement: S2 Fig — (A) Asymmetric PCR-digestion assay with template from enriched C. difficile R20291 flg ON and OFF isolates grown on an agar surface for 24 hours and 48 hours. T0 represents the cultures used to inoculate the agar plates. (B) Asymmetric PCR-digestion assay of C. difficile R20291 flg ON and OFF isolates grown in BHIS medium collected at T0, two exponential phase time points (EXP, OD600 0.5 and 1.0), and two stationary phase time points (STAT, OD600 1.8 and overnight, O/N). Images are representative from two independent experiments with four replicates of each flg phase. (C) Growth curve of C. difficile R20291 flg ON and OFF isolates in BHIS medium. Data are combined from two independent experiments each with two replicates of each flg phase, and means and standard deviations are shown. (PDF) [file pgen.1006701.s005.pdf]

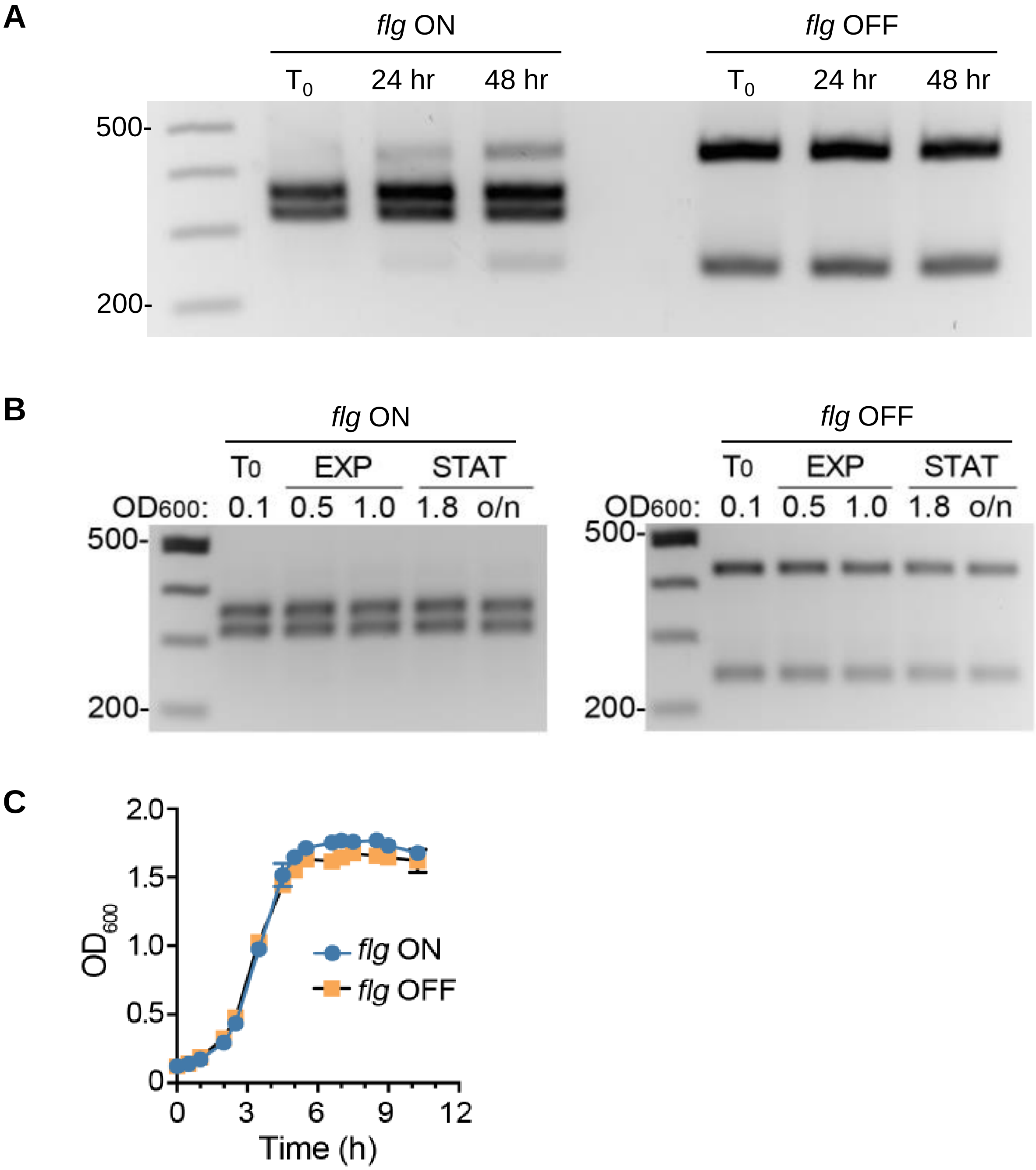

S2 Fig. Stability of the flagellar switch in enriched *flg* ON and OFF populations during growth in liquid and solid media. (A) Asymmetric PCR-digestion assay with template from enriched *C. difficile* R20291 *flg* ON and OFF isolates grown on an agar surface for 24 hours and 48 hours. T_0 represents the cultures used to inoculate the agar plates. (B) Asymmetric PCR-digestion assay of *C. difficile* R20291 *flg* ON and OFF isolates grown in BHIS medium collected at T_0, two exponential phase time points (EXP, OD_{600} 0.5 and 1.0), and two stationary phase time points (STAT, OD_{600} 1.8 and overnight, O/N). Images are representative from two independent experiments with four replicates of each *flg* phase. (C) Growth curve of *C. difficile* R20291 *flg* ON and OFF isolates in BHIS medium. Data are combined from two independent experiments each with two replicates of each *flg* phase, and means and standard deviations are shown.
