# Supplementary material for: A genetic switch controls the production of flagella and toxins in Clostridium difficile
Source: PLoS Genet. 2017 Mar 27;13(3):e1006701. doi: 10.1371/journal.pgen.1006701 (PMC5386303; doi:10.1371/journal.pgen.1006701)
Supplement: S3 Fig — C. difficile R20291 spores were plated on BHIS agar supplemented with taurocholate to obtain single colonies. After 24 hours, individual colonies were suspended in media and spotted onto fresh BHIS plates. Every 24 hours for four days, colony spots were suspended, serially diluted, and plated on BHIS to enumerate colonies based on their morphologies. (A) We observed two distinct colony morphologies based on colony texture and edge—a smooth, circular (SC) morphotype, and a rough, filamentous (RF) morphotype. (B) The percentage of RF colonies increased, and the percentage of SC colonies decreased over time. Using the asymmetric PCR-digestion assay to determine the orientation of the flagellar switch in SC and RF colonies after 96 hours, colony morphology and switch orientation were determined to be unlinked. 72% of bacteria in the SC colonies had the flagellar switch in the published orientation, and 64% in the RF colonies had the switch in the inverse orientation. (C,D) Bacteria from SC and RF colony morphology maintained their respective characteristic morphologies after passaging. (PDF) [file pgen.1006701.s006.pdf]

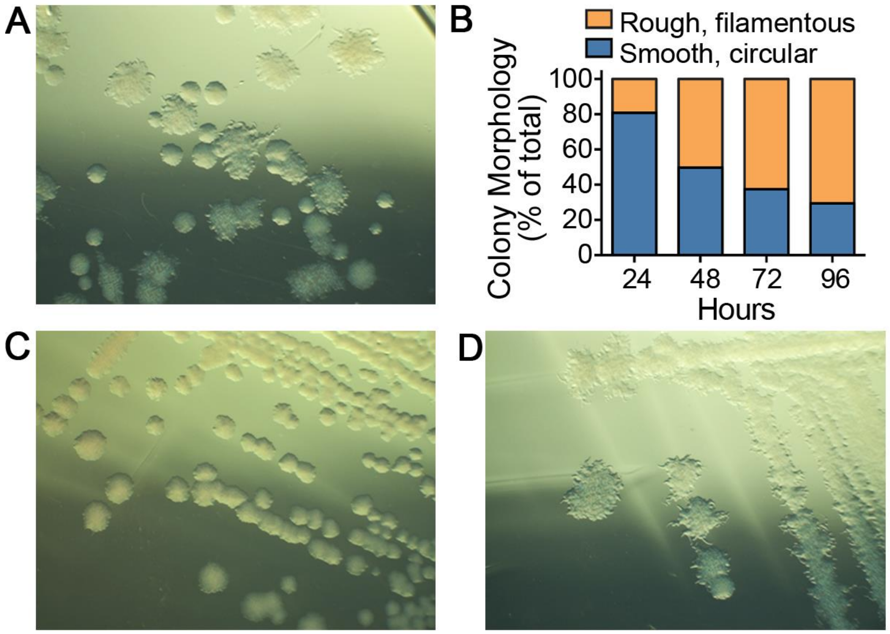

S3 Fig. Appearance of smooth, circular (SC) and rough, filamentous (RF) colony morphotypes. *C. difficile* R20291 spores were plated on BHIS agar supplemented with taurocholate to obtain single colonies. After 24 hours, individual colonies were suspended in media and spotted onto fresh BHIS plates. Every 24 hours for four days, colony spots were suspended, serially diluted, and plated on BHIS to enumerate colonies based on their morphologies. (A) We observed two distinct colony morphologies based on colony texture and edge– a smooth, circular (SC) morphotype, and a rough, filamentous (RF) morphotype. (B) The percentage of RF colonies increased, and the percentage of SC colonies decreased over time. Using the asymmetric PCR-digestion assay to determine the orientation of the flagellar switch in SC and RF colonies after 96 hours, colony morphology and switch orientation were determined to be unlinked. 72% of bacteria in the SC colonies had the flagellar switch in the published orientation, and 64% in the RF colonies had the switch in the inverse orientation. (C,D) Bacteria from SC and RF colony morphology maintained their respective characteristic morphologies after passaging.
