# Supplementary material for: A genetic switch controls the production of flagella and toxins in Clostridium difficile
Source: PLoS Genet. 2017 Mar 27;13(3):e1006701. doi: 10.1371/journal.pgen.1006701 (PMC5386303; doi:10.1371/journal.pgen.1006701)
Supplement: S4 Fig — (A) Frequency of RFP (mCherryOpt)-positive and -negative bacteria for strains evaluated in Fig 4D. Number of bacteria counted per group transformed with PflgM::mCherryOpt: flg ON (n = 3321 bacteria; 7 biological replicates), flg OFF (n = 3720 bacteria; 6 biological replicates), and sigD (n = 1348, 3 biological replicates). (B) Frequency of RFP (mCherryOpt)-positive and -negative bacteria of strains in panel C. Number of bacteria counted per group transformed with PTet::mCherryOpt: flg ON (n = 662 bacteria; 3 biological replicates) and flg OFF (n = 401 bacteria; 3 biological replicates). Number of bacteria counted per group transformed with promoterless::mCherryOpt: flg ON (n = 964 bacteria; 3 biological replicates), flg OFF (n = 772 bacteria; 3 biological replicates), and sigD (n = 670, 3 biological replicates). (C) Representative fluorescence micrographs for the indicated strains containing fusions of mCherryOpt to the Ptet promoter (grown with ATc, positive control) or promoterless mCherryOpt (negative controls). White bar = 10 microns. The channels used are indicated at the top. (PDF) [file pgen.1006701.s007.pdf]

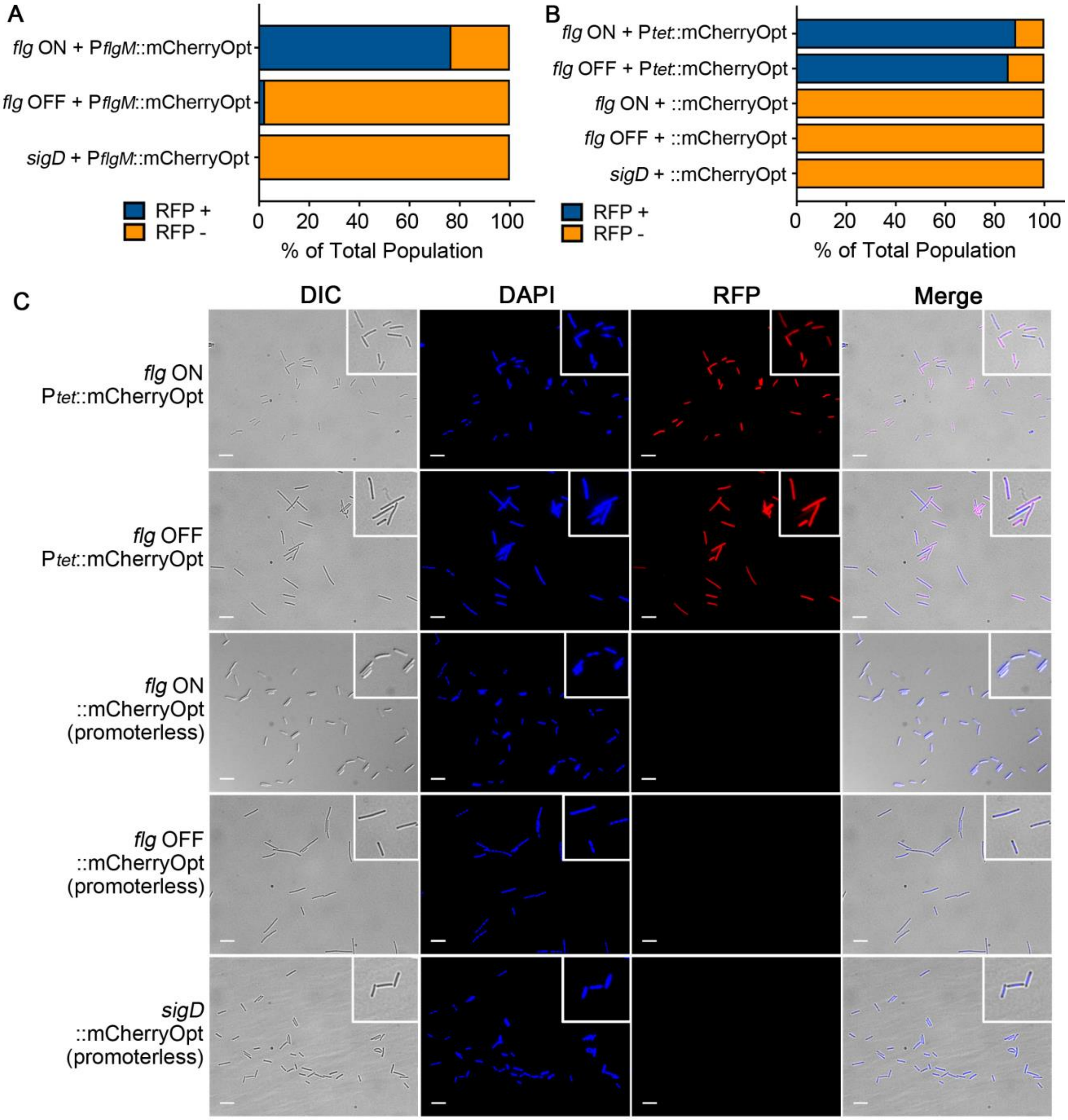

Figure S4. Quantification and controls for fluorescence microscopy studies in Fig 4. (A) Frequency of RFP (mCherryOpt)-positive and -negative bacteria for strains evaluated in Figure 4D. Number of bacteria counted per group transformed with P_{flgM}*::mCherryOpt*: *flg* ON (n=3321 bacteria; 7 biological replicates), *flg* OFF (n=3720 bacteria; 6 biological replicates), and *sigD* (n=1348, 3 biological replicates). (B) Frequency of RFP (mCherryOpt)-positive and -negative bacteria of strains in panel C. Number of bacteria counted per group transformed with P_{Tet}*::mCherryOpt*: *flg* ON (n=662 bacteria; 3 biological replicates) and *flg* OFF (n=401 bacteria; 3 biological replicates). Number of bacteria counted per group transformed with promoterless *::mCherryOpt*: *flg* ON (n=964 bacteria; 3 biological replicates), *flg* OFF (n=772 bacteria; 3 biological replicates), and *sigD* (n=670, 3 biological replicates). (C) Representative fluorescence micrographs for the indicated strains containing fusions of mCherryOpt to the P*tet* promoter (+ ATc, positive control) or promoterless mCherryOpt (negative controls). White bar = 10 microns. The channels used are indicated at the top.
