# Supplementary material for: A genetic switch controls the production of flagella and toxins in Clostridium difficile
Source: PLoS Genet. 2017 Mar 27;13(3):e1006701. doi: 10.1371/journal.pgen.1006701 (PMC5386303; doi:10.1371/journal.pgen.1006701)
Supplement: S5 Fig — (A). Schematic diagram of the Group II intron disruption of sigD in C. difficile R20291 (CDR20291_0270). The Targetron construct was designed previously to insert at nucleotide position 228 of the sigD gene in the sense orientation [60]. The sigD gene is 702 bp. The forward and reverse primers, R1887 (red arrow) and R1888 (green arrow), partially flank sigD and produce a PCR product of 716 bp. Insertion of the Group II intron into sigD (sigD::ermB) results in a PCR product of ~2500 bp. A second PCR reaction was used to confirm sigD::ermB by using R991, a Group II intron specific primer called EBS Universal. A PCR reaction with the R1887 and R991 yields a product of ~450 bp if the Group II intron is in sigD. (B). Image of an EtBr stained agarose gel with PCR products for the different reactions detailed in (A). Lane 1: Wildtype R20291 used as template with primers R1887 and R1888. Lane 2: The putative R20291 sigD::ermB mutant used as template with primers R1887 and R1888. Lane 3: The putative R20291 sigD::ermB mutant used as template with primers R1887 and R991. (PDF) [file pgen.1006701.s008.pdf]

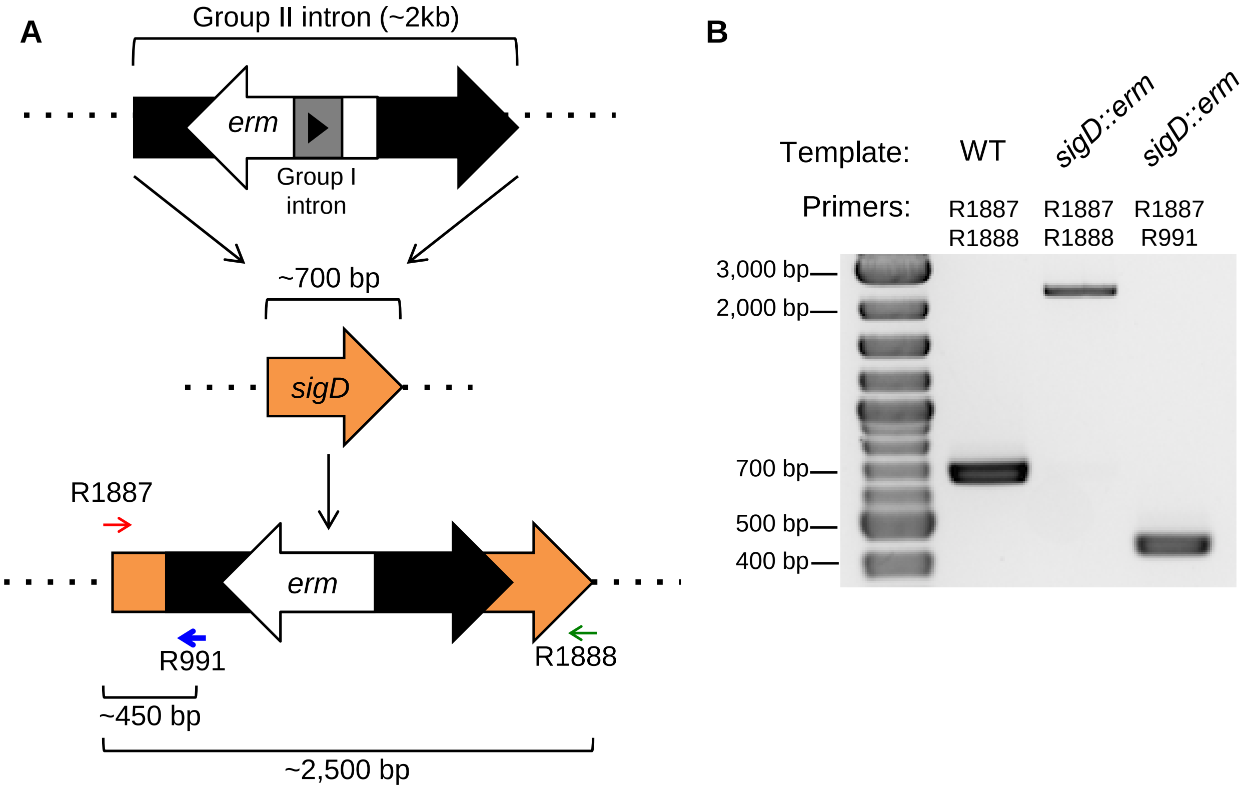

S5 Fig. Construction and confirmation of the *sigD::ermB* mutation in *C. difficile* R20291. (A) Schematic diagram of the Group II intron disruption of *sigD* in *C. difficile* R20291 (CDR20291_0270). The Targetron construct was designed previously to insert at nucleotide position 228 of the *sigD* gene in the sense orientation. The *sigD* gene is 702 bp. The forward and reverse primers, R1887 (red arrow) and R1888 (green arrow), partially flank *sigD* and produce a PCR product of 716 bp. Insertion of the Group II intron into *sigD* (*sigD::ermB*) results in a PCR product of ~2500 bp. A second PCR reaction was used to confirm *sigD::ermB* by using R991, a Group II intron specific primer called EBS Universal. A PCR reaction with the R1887 and R991 yields a product of ~450 bp if the Group II intron is in *sigD*. (B) Image of an EtBr stained agarose gel with PCR products for the indicated strains with primers detailed in (A).
