# Supplementary material for: A genetic switch controls the production of flagella and toxins in Clostridium difficile
Source: PLoS Genet. 2017 Mar 27;13(3):e1006701. doi: 10.1371/journal.pgen.1006701 (PMC5386303; doi:10.1371/journal.pgen.1006701)
Supplement: S6 Fig — (A, B) Asymmetric PCR-digestion assay to determine the orientation of the flagellar switch. For the native locus, we used primers R1705 and R1704 in PCR reactions. R1705 (forward primer) anneals 3’ of the Cd1 riboswitch DNA sequence, and R1704 (reverse primer) anneals to the second gene in the flgB operon, flgC (CDR20291_0249). For the reporter locus (B), we used R1705 and R1706, a reverse primer that anneals to the alkaline phosphatase gene, phoZ, for PCR amplification. Biological replicates for each AP reporter in C. difficile R20291 were combined and genomic DNA was extracted, based on published methods, to simultaneously determine the orientation of the native flagellar switch in each. The numerical designations for each reporter are shown in parentheses and correspond to Fig 7A. We confirmed that both the native and reporter flagellar switches for each AP reporter strain were in their expected orientations. (C) Orientation-specific PCR assay of AP reporters #3–6 in B. subtilis showing that the AP reporter flagellar switches maintained their orientations. (PDF) [file pgen.1006701.s009.pdf]

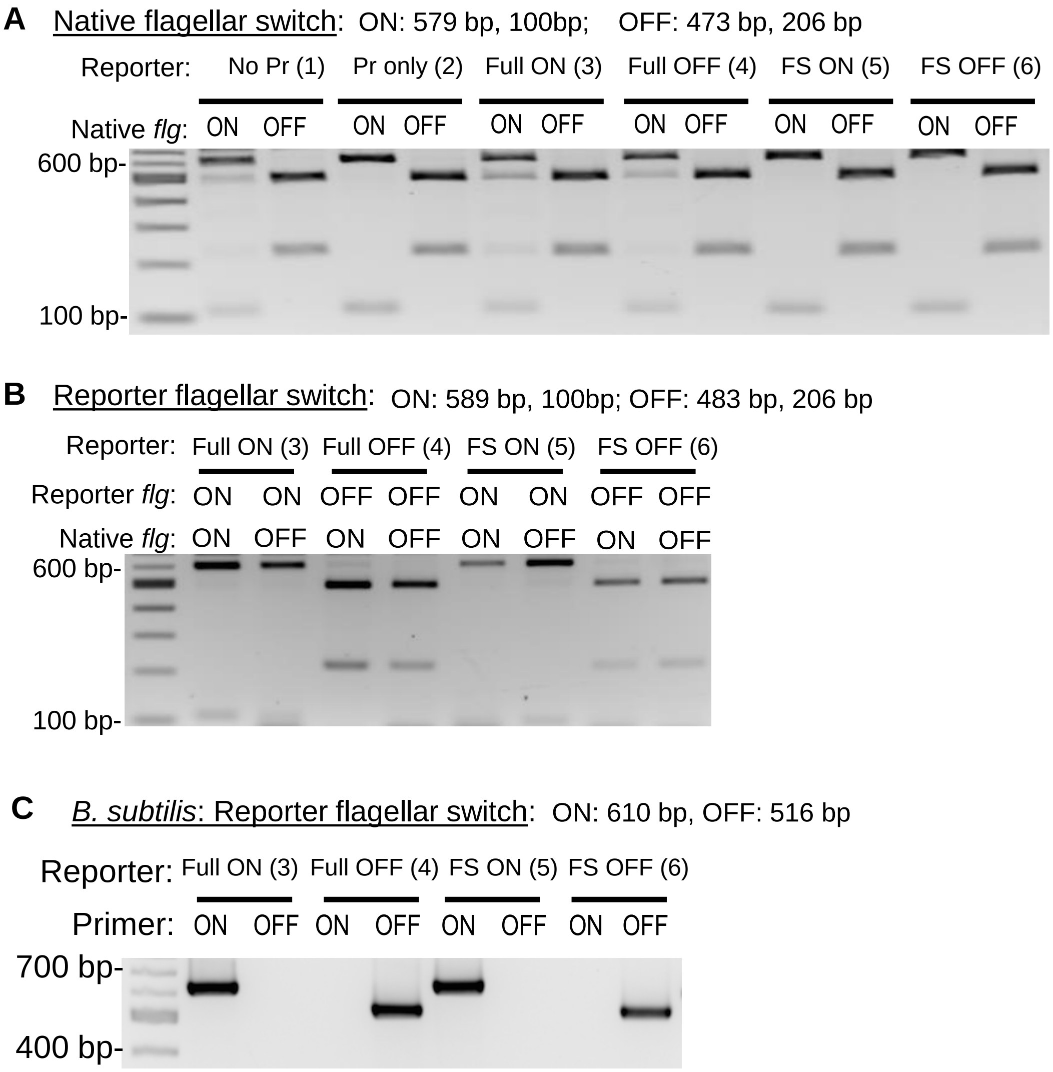

S6 Fig. Confirmation of flagellar switch orientation accompanying alkaline phosphatase assays in *C. difficile* and *B. subtilis*. (A, B) Asymmetric PCR-digestion assay to determine the orientation of the flagellar switch. For the native locus, we used primers R1705 and R1704 in PCR reactions. R1705 (forward primer) anneals 3’ of the Cd1 riboswitch DNA sequence, and R1704 (reverse primer) anneals to the second gene in the *flgB* operon, *flgC* (CDR20291_0249). For the reporter locus (B), we used R1705 and R1706, a reverse primer that anneals to the alkaline phosphatase gene, *phoZ*, for PCR amplification. Biological replicates for each AP reporter in *C. difficile* R20291 were combined and genomic DNA was extracted, based on published methods, to simultaneously determine the orientation of the native flagellar switch in each. The numerical designations for each reporter are shown in parentheses and correspond to Fig 7A. We confirmed that both the native and reporter flagellar switches for each AP reporter strain were in their expected orientations. (C) Orientation-specific PCR assay of AP reporters #3-6 in *B. subtilis* showing that the AP reporter flagellar switches maintained their orientations.
