# Supplementary material for: A genetic switch controls the production of flagella and toxins in Clostridium difficile
Source: PLoS Genet. 2017 Mar 27;13(3):e1006701. doi: 10.1371/journal.pgen.1006701 (PMC5386303; doi:10.1371/journal.pgen.1006701)
Supplement: S7 Fig — Activity was assessed as described in the main text in tandem with the same reporters in the R20291 flg ON background. (PDF) [file pgen.1006701.s010.pdf]

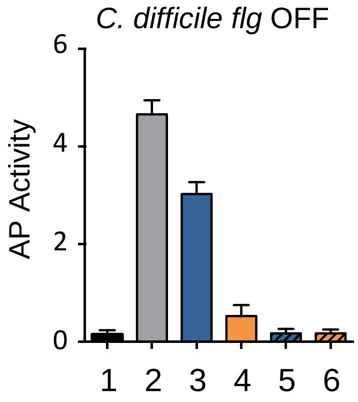

S7 Fig. Alkaline phosphatase activity of the *phoZ* gene reporters in *C. difficile flg* OFF. Activity was assessed as described in the main text in parallel with the same reporters in the R20291 *flg* ON background.
