# Supplementary material for: A genetic switch controls the production of flagella and toxins in Clostridium difficile
Source: PLoS Genet. 2017 Mar 27;13(3):e1006701. doi: 10.1371/journal.pgen.1006701 (PMC5386303; doi:10.1371/journal.pgen.1006701)
Supplement: S8 Fig — (A) Asymmetric PCR-digestion assay of products from C. difficile R20291 flg ON and OFF isolates transformed with pRT1611 (V, vector), or pRT1529 (pRPF185::recV), grown with or without 10 ng/ml ATc (S1 Table). (B) Increased transcription of recV occurs in C. difficile with pRT1529 in the absence of ATc induction. qRT-PCR measuring transcript abundance of recV and codY control gene in R20291 flg ON and OFF, each with pRT1611 (vector) and RT1529 (recV). Data were normalized to the rpoC gene and expressed relative to the respective parental strain with pRT1611. Shown are means and standard deviations. (PDF) [file pgen.1006701.s011.pdf]

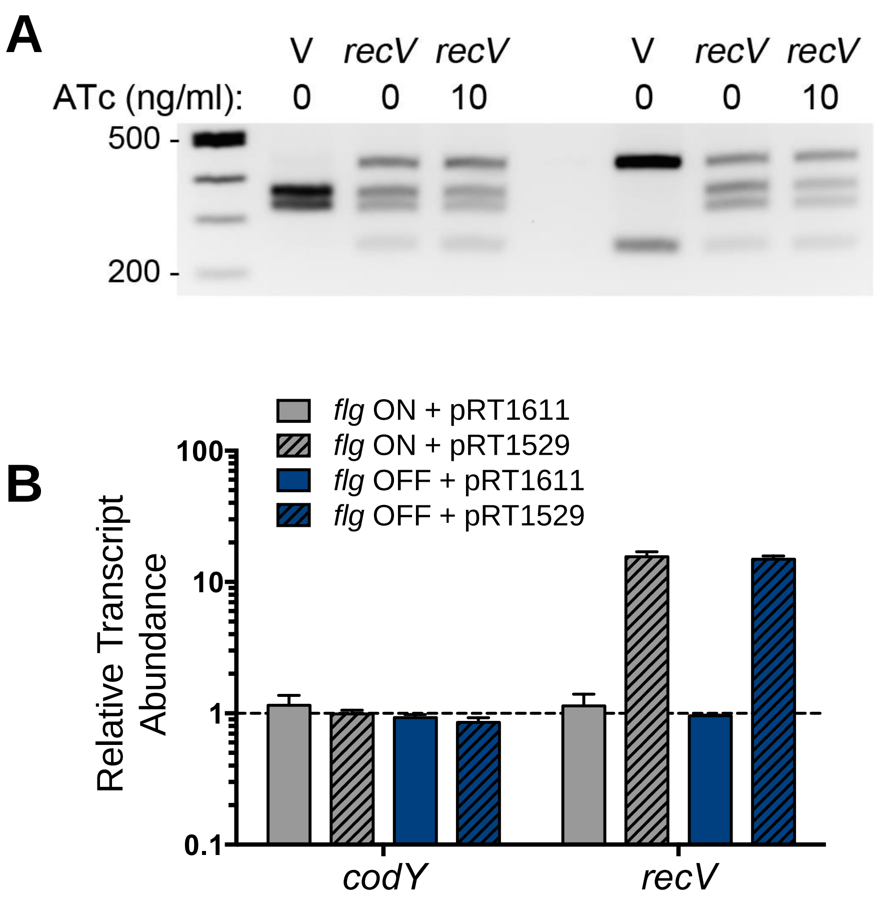

S8 Fig. Expression of *recV* in *C. difficile flg* ON and *flg* OFF isolates stimulates inversion of the flagellar switch. (A) Asymmetric PCR-digestion assay of products from *C. difficile* R20291 *flg* ON and OFF isolates transformed with pRT1611 (V, vector), or pRT1529 (pRPF185::*recV*), grown with or without 10 ng/ml ATc (S1 Table). (B) Increased transcription of *recV* occurs in *C. difficile* with pRT1529 in the absence of ATc induction. qRT-PCR measuring transcript abundance of *recV* and *codY* control gene in R20291 *flg* ON and OFF, each with pRT1611 (vector) and RT1529 (*recV*). Data were normalized to the *rpoC* gene and expressed relative to the respective parental strain with pRT1611. Shown are means and standard deviations.
