# Supplementary material for: A genetic switch controls the production of flagella and toxins in Clostridium difficile
Source: PLoS Genet. 2017 Mar 27;13(3):e1006701. doi: 10.1371/journal.pgen.1006701 (PMC5386303; doi:10.1371/journal.pgen.1006701)
Supplement: S9 Fig — C. difficile R20291 recV flg OFF* strain was transformed with a plasmid for expression of recV to allow flagellar switch inversion. The strain was then passaged without antibiotics to allow plasmid loss. Five thiamphenicol-sensitive colonies were identified and screened by PCR as in Fig 1 for the orientation of the cwpV and flagellar switches. Shown: orientation-specific PCR assay of five isolates, two of which (#1, #3) have the flagellar switch in the ON orientation; both have the cwpV switch in the OFF orientation. (PDF) [file pgen.1006701.s012.pdf]

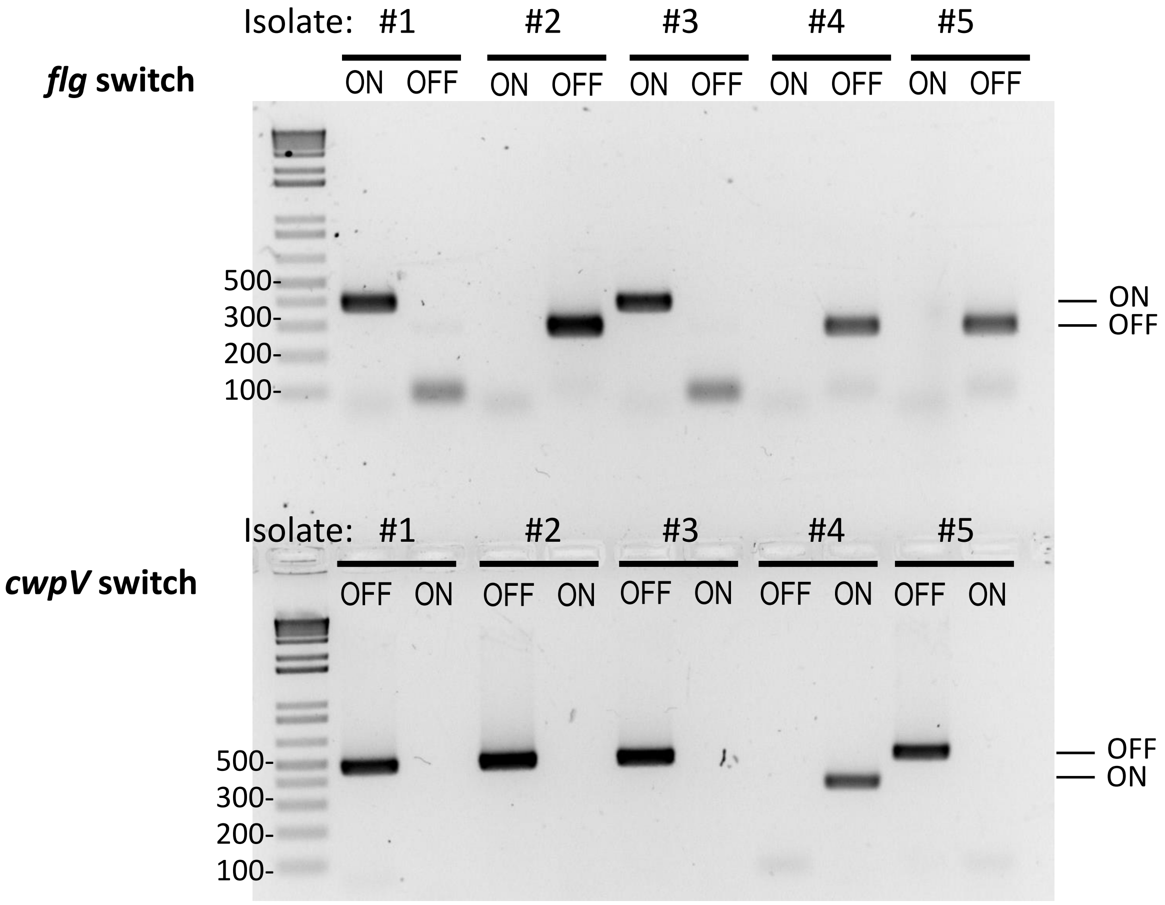

S9 Fig. Identification of *C. difficile* R20291 *recV flg* ON* mutants. *C. difficile* R20291 *recV flg* OFF* was transformed with a plasmid for expression of *recV* to allow flagellar switch inversion. The strain was then passaged without antibiotics to allow plasmid loss. Five thiamphenicol-sensitive colonies were identified and screened by PCR as in Figure 1 for the orientation of the *cwpV* and flagellar switches. Shown: orientation-specific PCR assay of five isolates, two of which (#1, #3) have the flagellar switch in the ON orientation; both have the *cwpV* switch in the OFF orientation.
