# Supplementary material for: A genetic switch controls the production of flagella and toxins in Clostridium difficile
Source: PLoS Genet. 2017 Mar 27;13(3):e1006701. doi: 10.1371/journal.pgen.1006701 (PMC5386303; doi:10.1371/journal.pgen.1006701)
Supplement: S10 Fig — (A) Representative image of a motility assay after 72 hours, with flg ON/pPtet (RT1615), flg OFF/pPtet (RT1617), sigD/pPtet (RT1690), recV flg ON*/pPtet (RT1715), recV flg ON*/pPtet::recV (RT1716), recV flg OFF*/pPtet (RT1691), and recV flg OFF*/pPtet::recV (RT1697). Strain numbers are listed in parentheses. The recV flg OFF*/pPtet::recV (RT1691) showed motility upon this prolonged incubation. (B) Orientation-specific PCR assay of the flagellar switch from recV flg ON*, recV flg OFF*, and three motile suppressor (MS) mutants of recV flg OFF* (pRT1719 –RT1724). Image representative of two independent experiments with eight biological replicates of motile suppressor mutants of recV flg OFF*. (PDF) [file pgen.1006701.s013.pdf]

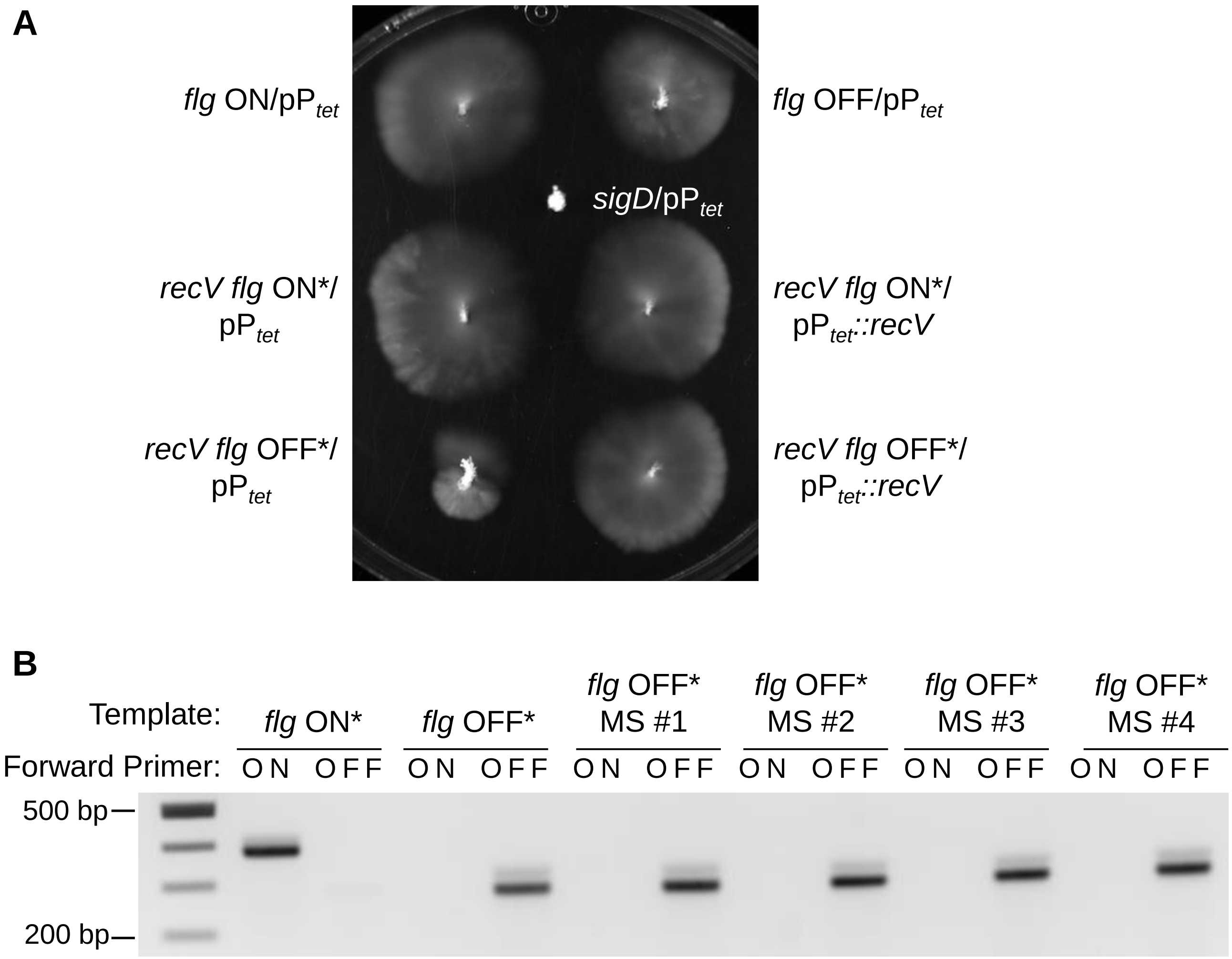

S10 Fig. Isolation of motile *recV flg* OFF* suppressor mutants. (A) Representative image of a motility assay after 72 hours, with *flg* ON/pP$_{tet}$ (RT1615), *flg* OFF/pP$_{tet}$ (RT1617), *sigD*/pP$_{tet}$ (RT1690), *recV flg* ON*/pP$_{tet}$ (RT1715), *recV flg* ON*/pP$_{tet}$::*recV* (RT1716), *recV flg* OFF*/pP$_{tet}$ (RT1691), and *recV flg* OFF*/pP$_{tet}$::*recV* (RT1697). Strain numbers are listed in parentheses. The *recV flg* OFF*/pP$_{tet}$::*recV* (RT1691) showed motility upon this prolonged incubation. (B) Orientation-specific PCR assay of the flagellar switch from *recV flg* ON*, *recV flg* OFF*, and three motile suppressor (MS) mutants of *recV flg* OFF* (pRT1719 – RT1724). Image representative of two independent experiments with eight biological replicates of motile suppressor mutants of *recV flg* OFF*.
