# Supplementary material for: A genetic switch controls the production of flagella and toxins in Clostridium difficile
Source: PLoS Genet. 2017 Mar 27;13(3):e1006701. doi: 10.1371/journal.pgen.1006701 (PMC5386303; doi:10.1371/journal.pgen.1006701)
Supplement: S11 Fig — The following C. difficile genomes were used in a sequence alignment for the flagellar switch: 630, BI9, M68, CF5, R20291, CD196, BI1, and 2007855. (PDF) [file pgen.1006701.s014.pdf]

```
630          AAGTTACTATTTTACAAAAA-  GAGCAACTTTTCGAAGAAATATTTAAATACATATTAAAATTTTT  64
BI9          AAGTTACTATTTTACAAAAAA  GAGCAACTTTTCGAAGAAATATTTAAATACATATTAAAATTTTT  65
M68          AAGTTACTATTTTACAAAAAA  GAGCAACTTTTTGAAGAAATATTTAAATACATATTAAAATTTTT  65
CF5 Inverse  AAGTTACTATTTTACAAAAAA  GAGCAACTTTTTGAAGAAATATTTAAATACATATTAAAATTTTT  65
R20291       AAGTTACTATTTTACAAAAAA  AGGCAACTTTATAAAGAAATATTTAAATTTATATTAAAATATTT  65
CD196        AAGTTACTATTTTACAAAAAA  AGGCAACTTTATAAAGAAATATTTAAATTTATATTAAAATATTT  65
BI1 Inverse  AAGTTACTATTTTACAAAAAA  AGGCAACTTTATAAAGAAATATTTAAATTTATATTAAAATATTT  65
2007855      AAGTTACTATTTTACAAAAAA  AGGCAACTTTATAAAGAAATATTTAAATTTATATTAAAATATTT  65

630          TTATATTTTCATGGGACTTCATTAAAAAAATATTAGTTTCTTACTAAATTGATACATTATTGTATT  131
BI9          TTATATTTTCATGGGACTTCATTGAAAAAATATTAGTTTCTTACTAAAATGATACATTATTGTATT  132
M68          TTATATTTTCATGGGACTTCATTGAAAAAATATTAGTTTCTTACTAAAATGATACATTATTGTATT  132
CF5 Inverse  TTATATTTTCATGGGACTTCATTGAAAAAATATTAGTTTCTTACTAAAATGATACATTATTGTATT  132
R20291       TTATATTTTTATTAGGATTTACGAAAATAATATTAGTTTCTTACCAAAGTGATACATTATTATATT  132
CD196        TTATATTTTTATTAGGATTTACGAAAATAATATTAGTTTCTTACCAAAGTGATACATTATTATATT  132
BI1 Inverse  TTATATTTTTATTAGGATTTACGAAAATAATATTAGTTTCTTACCAAAGTGATACATTATTATATT  132
2007855      TTATATTTTTATTAGGATTTACGAAAATAATATTAGTTTCTTACCAAAGTGATACATTATTATATT  132

630          AATGTAATGGAAATGCGATTAATTTAAATAAATAAAGAAGTCA  TT-TTTGTAATATAGCAACTT  195
BI9          AATGTAATGGAAATGCGATTAATTTAAATGAATAAAGAAGTCA  TTTTTTGTAATATAGCAACTT  196
M68          AATGTAATGGAAATGCGATTAATTTAAATGAATAAAGAAGTCA  TTTTTTGTAATATAGCAACTT  196
CF5 Inverse  AATGTAATGGAAATGCGATTAATTTAAATGAATAAAGAAGTCA  TTTTTTGTAATATAGCAACTT  196
R20291       AATGTAATAGAAATGTAATTAATTTGGATGAATAAAGAAGTCA  TTTTTTGTAATATAGCAACTT  196
CD196        AATGTAATAGAAATGTAATTAATTTGGATGAATAAAGAAGTCA  TTTTTTGTAATATAGCAACTT  196
BI1 Inverse  AATGTAATAGAAATGTAATTAATTTGGATGAATAAAGAAGTCA  TTTTTTGTAATATAGCAACTT  196
2007855      AATGTAATAGAAATGTAATTAATTTGGATGAATAAAGAAGTCA  TTTTTTGTAATATAGCAACTT  196
```

S11 Fig. Sequence alignment of the flagellar switch and inverted repeat sequences from NCBI accessible genomes of *C. difficile*. The following *C. difficile* genomes were used in a sequence alignment with Clustal Omega for the flagellar switch: 630, BI9, M68, CF5, R20291, CD196, BI1, and 2007855.
